# VIETNAMESE POLITICAL CULTURE: STRUCTURE, ROLE, AND DEVELOPMENT ORIENTATION IN THE NEW ERA

**DOI:** 10.12688/f1000research.177771.1

**Published:** 2026-05-07

**Authors:** Pham Thi Thuy Van, LAI QUOC KHANH

**Affiliations:** 1 Ha Noi Pedagogical University 2, Phuc Yen, Vietnam; 2 Ho Chi Minh National Academy of Politics, Hanoi, Vietnam

**Keywords:** Vietnamese political culture; Political values; Governance culture; Sustainable development; Renovation

## Abstract

This article examines the structure and role of Vietnamese political culture in the context of the country’s new stage of development, with reference to the orientations set out in the Draft Political Report submitted to the Fourteenth National Congress of the Communist Party of Vietnam. Applying an interdisciplinary approach that integrates Marxist–Leninist theory, Ho Chi Minh Thought, and contemporary political culture studies, the article conceptualizes political culture not only as a spiritual foundation but also as an internal resource, a soft regulatory mechanism, and a driving force for sustainable development. On this basis, the study proposes a three-tier analytical framework of Vietnamese political culture, encompassing values and ideology, norms and political behavior, and institutions and governance culture. Drawing on a synthesis of theoretical perspectives and an analysis of Vietnam’s Renovation experience from 1986 to 2025, the article clarifies the dialectical relationship between political culture, institutional quality, governance effectiveness, and social trust. The findings indicate that Vietnamese political culture is undergoing a gradual transformation from a mobilization-oriented model to a development- and creativity-oriented model, reflecting the interplay among national traditions, socialist ideals, and modern governance principles. The article also outlines policy implications for institutionalizing political-culture values, strengthening political culture within governance institutions, and supporting Vietnam’s development goals through 2030 and the strategic vision for 2045.

## I. Introduction

In the context of globalization, digital transformation, and deep international integration, political culture is increasingly recognized as a foundational determinant of sustainable development. It is not merely a component of the political superstructure but also functions as a soft regulatory system and an endogenous resource shaping political cognition, behavior, institutional performance, and national identity. ^1^ Contemporary development can no longer be assessed solely through economic growth indicators; rather, it must also be evaluated in terms of institutional quality, governance capacity, transparency, innovation, and social trust, dimensions that directly reflect the maturity of political culture. ^2^ In this sense, political culture constitutes both the normative infrastructure and the adaptive capacity of modern states.

In Vietnam, the significance of political culture has become particularly salient as the Communist Party of Vietnam prepares for its Fourteenth National Congress. The Draft Political Report submitted to the Congress affirms a breakthrough awareness: culture and human beings are not only the spiritual foundation of society but also internal resources, regulatory systems, and driving forces of sustainable development. ^3^ This represents an important evolution in political thinking, from viewing culture as a supportive sphere of socio-economic development to recognizing it as a central determinant of development quality and national resilience.

The urgency of studying Vietnamese political culture arises from both theoretical and practical considerations. Classical scholarship on political culture, especially the foundational works of Almond and Verba ^4^ and Pye and Verba, ^5^ established the relationship between civic orientations and democratic stability. Subsequent research has further emphasized the role of trust, social capital, ^6^ and institutional effectiveness in sustaining governance systems. ^7^ However, these theoretical models were largely formulated within Western liberal democratic contexts and may not fully account for the structural and ideological characteristics of socialist-oriented and developing societies, where political culture is closely intertwined with collective value systems and the leadership role of the ruling party. In contrast, Marxism–Leninism and Ho Chi Minh Thought conceptualize politics as inseparable from morality and human development. ^8^ Ho Chi Minh consistently affirmed that political leadership must be grounded in revolutionary ethics and regarded culture as a guiding force illuminating the path of national development. ^9^ Thus, examining Vietnamese political culture today is not only an act of theoretical inheritance but also a necessary effort to develop Marxist–Leninist and Ho Chi Minh thought under contemporary conditions.

From a practical perspective, nearly a century since the founding of the Communist Party of Vietnam and four decades of Renovation ( *Đổi Mới*) have generated profound transformations in Vietnam’s political and social life. ^1^ The country has achieved notable progress in economic growth, innovation performance, ^10^ digital transformation, and international integration. ^11^ At the same time, new challenges have emerged, including concerns regarding public service ethics, ^2^ governance transparency, behavioral norms in digital spaces, and the maintenance of social trust. ^12^ The development of a socialist-oriented market economy and the intensification of global integration, combined with the pressures of the Fourth Industrial Revolution, require a restructuring of political culture along the principles of integrity, accountability, creativity, and civic responsibility. Without a resilient political culture, modernization processes risk generating value dislocation, erosion of trust, and identity fragmentation.

Within global scholarly discourse, ongoing crises of confidence in liberal democratic systems and the rise of populist movements have renewed attention to alternative governance models. ^7^ Some scholars have highlighted the relevance of East Asian political-cultural configurations characterized by strong state capacity, ^6^ communitarian values, and ethical public administration. ^13^ Vietnam’s political culture, shaped by national traditions, socialist ideals, and adaptive integration, offers a distinctive case for comparative analysis in this evolving debate.

Against this backdrop, the present article seeks to systematize the theoretical foundations and analyze the structure and developmental role of Vietnamese political culture in the contemporary period. Drawing on four decades of Renovation practice, the study identifies key dynamics of transformation and proposes orientations toward the 2045 development horizon. Grounded in Marxism–Leninism, Ho Chi Minh Thought, and the Party’s contemporary perspectives on culture and human development, the article conceptualizes political culture as a dynamic and dialectical unity of values, behavioral norms, and institutional structures that collectively generate the nation’s spiritual strength and developmental momentum.

## II. Theoretical overview and ideational foundations

The concept of political culture emerged as a central analytical category in comparative political science during the mid-twentieth century. It was systematically articulated in *The Civic Culture* by Almond and Verba, who defined political culture as a pattern of subjective orientations toward political action, encompassing citizens’ attitudes, beliefs, values, and competencies in relation to the political system and their roles within it. ^4^ This formulation marked a significant departure from purely institutional analyses of politics by emphasizing the psychological and normative dimensions underpinning political stability and democratic performance. Expanding this perspective, Pye conceptualized political culture as the total configuration of shared meanings, symbols, and value orientations through which individuals and groups define political roles and legitimate authority. ^5^ In this view, political culture operates not merely as individual disposition but as a collective interpretive framework structuring political life.

Subsequent scholarship diversified the theoretical landscape. Inglehart advanced a modernization-based interpretation, arguing that socio-economic development generates a gradual shift from materialist priorities focused on economic and physical security toward post-materialist values emphasizing autonomy, participation, and self-expression. ^14^ This transformation, he suggested, reshapes political culture and alters the foundations of legitimacy and governance. Building on this line of inquiry, Welzel proposed that emancipative values, centered on individual choice and equal opportunity, constitute an internal cultural force sustaining democratic development and institutional accountability. ^15^ From a complementary institutional perspective, Putnam demonstrated that civic traditions and social capital significantly influence governmental performance, illustrating the reciprocal relationship between cultural norms of trust and the effectiveness of public institutions. ^7^ Together, these approaches converge on the understanding that political culture constitutes a system of collectively shared values and behavioral expectations that mediates the relationship between citizens and the state, thereby shaping governance outcomes.

Despite their analytical sophistication, these theoretical models were largely developed within Western liberal democratic contexts. Their underlying assumption pluralist competition, individualist value systems, and electoral accountability, may not fully capture the structural and ideological configurations of socialist-oriented or developing societies. In such contexts, political culture is closely intertwined with collective value orientations, national traditions, and the guiding role of a ruling party in directing socio-economic transformation. The normative and institutional foundations of political legitimacy may therefore differ substantially from those assumed in liberal-democratic paradigms.

Within the Vietnamese context, the ideational foundations of political culture are deeply rooted in Marxism–Leninism and Ho Chi Minh Thought. Marxist theory conceives politics and culture as dialectically interrelated dimensions of social development, wherein moral consciousness and material conditions mutually shape historical transformation. Ho Chi Minh further elaborated this relationship by emphasizing the ethical grounding of political leadership ^8^ and the formative role of culture in national development. ^9^ For Ho Chi Minh, revolutionary morality constituted the ethical core of political authority, while culture functioned as a guiding light for societal progress. This perspective situates political culture not merely as attitudinal orientation but as a moral–ideological system embedded in institutional practice and collective identity.

Accordingly, the study of Vietnamese political culture requires a theoretical synthesis that moves beyond the dichotomy between Western civic-cultural models and purely institutional analyses. It calls for an approach that integrates value systems, ideological foundations, and governance structures within a dialectical framework. Such an approach recognizes political culture as a dynamic configuration of values, norms, and institutional practices that evolve in response to socio-economic transformation while maintaining continuity with foundational ideological principles.

## III. Research design and methodology

This study adopts a qualitative and interdisciplinary research design to examine the structure and transformation of Vietnamese political culture during the Renovation ( *Đổi Mới*) period from 1986 to 2025. The research is conceptual, interpretive, and analytical in orientation. It does not seek to test causal hypotheses through primary data collection; rather, it develops a theoretically grounded framework for understanding the evolution of political culture in relation to socio-economic transformation and governance reform. The study synthesizes political theory analysis, historical–institutional inquiry, and secondary quantitative interpretation to construct a coherent analytical model of contemporary Vietnamese political culture.

The analytical framework is structured around three interrelated dimensions: foundational ideological orientations, norms and patterns of political behavior, and institutional and governance culture. This tripartite structure functions as a heuristic device for organizing both theoretical reflection and empirical illustration. It enables systematic examination of the interaction between ideological continuity and adaptive governance practices throughout the reform era, while situating Vietnam’s experience within broader debates on political culture and modernization.

The study relies exclusively on documentary analysis and secondary data sources. First, it engages in qualitative content analysis of foundational ideological texts and official documents, including classical Marxist–Leninist works, the writings of Ho Chi Minh, and resolutions and policy documents issued by the Communist Party of Vietnam across successive Party Congresses. These texts are examined to identify recurring conceptual themes, normative principles, and institutional orientations shaping political culture. Second, the research incorporates publicly available secondary statistical indicators and international datasets to contextualize long-term trends in governance performance, human development, innovation capacity, and public trust. These indicators are used descriptively to illustrate structural transformation over time rather than to perform econometric modeling or primary statistical testing.

No primary empirical research involving human participants, such as surveys, interviews, focus groups, or experimental interventions, was conducted for the purposes of this study. All data utilized are derived from publicly accessible documents, official publications, and international datasets that are already in the public domain. Accordingly, the research does not involve human subjects, does not collect personal or identifiable information, and does not require ethical approval or informed consent under national or international research ethics guidelines for studies involving human participants. The study is therefore exempt from institutional review board oversight as it constitutes documentary and secondary data analysis within the fields of political theory and social science research.

Methodologically, the research combines qualitative content analysis, historical process tracing, and descriptive interpretation of secondary indicators. Triangulation across ideological texts, institutional documents, and publicly available statistical sources enhances analytical coherence and interpretive robustness. The temporal scope spans from the initiation of *Đổi Mới* reforms in 1986 to 2025, enabling longitudinal assessment of both continuity and transformation in Vietnamese political culture. As an interpretive and theory-building inquiry grounded in documentary and secondary evidence, the findings should be understood as analytical contributions to debates on political culture and governance transformation rather than as causal determinations derived from primary empirical testing.

## IV. Results and discussion

### 1. The marxist–leninist and ho chi minh thought approaches to political culture

From a Marxist–Leninist perspective, political culture cannot be understood as a peripheral or purely symbolic phenomenon; rather, it constitutes a dynamic component of the broader superstructural formation that both reflects and shapes material conditions. Marx’s foundational proposition that the economic base conditions the political and ideological superstructure has often been interpreted deterministically; however, contemporary scholarship underscores that Marx simultaneously recognized the relative autonomy and transformative capacity of ideology once internalized by social actors. ^16^ In this sense, political culture operates dialectically: it emerges from socio-economic relations yet becomes a material force when embedded in collective consciousness and organized political practice. Political culture, therefore, is not reducible to institutional form but must be analyzed as a mediating layer through which structural conditions are interpreted, legitimized, and contested.

Lenin’s theoretical development further clarified this mediating function. His conception of political consciousness emphasized the necessity of organized ideological education in transforming spontaneous class awareness into coherent political agency. ^17^ Political culture, in this view, is neither spontaneous nor culturally neutral; it is cultivated through disciplined organization, moral commitment, and collective purpose. Recent reinterpretations of Leninist political theory suggest that what distinguishes the Leninist model is its fusion of ethical rigor and institutional discipline, whereby legitimacy derives not only from formal authority but from demonstrable alignment with mass interests and historical mission. ^18^ Consequently, political culture within a Marxist–Leninist framework functions as an integrative mechanism that binds leadership, ideology, and popular mobilization into a coherent political project.

Ho Chi Minh’s contributions represent both continuity and contextual innovation within this tradition. Rather than treating culture as subordinate to politics, he conceptualized culture as an orienting force that shapes the ethical and developmental trajectory of the nation. Contemporary analyses of Ho Chi Minh Thought emphasize its synthesis of Marxist theory with Vietnamese historical traditions and Confucian moral philosophy, producing a distinctive moral-political framework centered on public virtue, collective solidarity, and national independence. ^19^ In this framework, political legitimacy is inseparable from moral character. Leadership competence is evaluated not solely through administrative efficiency but through integrity, self-discipline, and devotion to the public good. Political culture thus becomes embodied in ethical conduct and everyday governance practice.

Analytically, Ho Chi Minh’s approach reframes political culture as a triadic structure comprising popular orientation, ethical foundation, and openness to civilizational exchange. First, political culture is human-centered, affirming that state power must ultimately serve the people and derive legitimacy from their trust. Second, it is grounded in moral discipline, emphasizing integrity, thrift, impartiality, and responsibility as conditions of sustainable authority. Third, it remains outward-looking and adaptive, capable of integrating global knowledge while preserving national identity. Recent scholarship on Vietnam’s political development suggests that this ethical–institutional synthesis has contributed to regime resilience and governance stability during periods of rapid economic transformation. ^20^

Taken together, the Marxist–Leninist and Ho Chi Minh perspectives conceptualize political culture not as a passive reflection of structural forces but as a normative and organizational resource. It mediates between ideology and institution, morality and authority, leadership and mass participation. Within the Vietnamese context, this ideational foundation provides a framework through which political modernization is pursued without severing continuity with revolutionary ethics and collective developmental goals. Political culture thus functions simultaneously as soft normative power and as an internal stabilizing mechanism in the country’s ongoing Renovation and international integration processes.

### 2. An integrated approach: connecting marxism–leninism, ho chi minh thought, and modern theory

The comparative analysis of political culture reveals both divergence and convergence between Western civic-cultural theory and the Marxist–Leninist–Ho Chi Minh tradition. Western scholarship has historically emphasized citizens’ beliefs, participatory norms, ^4^ and civic behavior as determinants of democratic stability. ^7^ Within this framework, political culture is often conceptualized as a bottom-up phenomenon that conditions institutional effectiveness through trust, social capital, and value orientations. By contrast, Marxism–Leninism and Ho Chi Minh Thought foreground the ethical foundations of political authority and the legitimacy of power as central components of political culture. Rather than focusing primarily on individual attitudes, this tradition conceptualizes political culture as a morally structured relationship between leadership and the people, mediated by ideological coherence and collective purpose.

Despite these differences in emphasis, contemporary comparative research suggests that the dichotomy between “Western” and “non-Western” models is increasingly untenable. Fukuyama’s analysis of political order argues that sustainable development depends not solely on electoral competition but on the interplay between state capacity, rule-based governance, and cultural norms that reinforce institutional discipline and public trust. ^6^ His assessment of East Asian developmental trajectories underscores the importance of communitarian values and administrative integrity, traits that resonate with elements of Marxist–Leninist political ethics. Similarly, Inglehart and Welzel’s revised modernization theory acknowledges that democratic quality is shaped by culturally embedded value systems and need not replicate Western historical pathways, if governance institutions maintain effectiveness and public legitimacy. ^21^ Political culture, therefore, emerges as a mediating variable across diverse developmental models, linking normative orientations with institutional outcomes.

Situated within this evolving theoretical landscape, Vietnam represents a distinctive case in which Marxist–Leninist principles, Ho Chi Minh’s moral–political philosophy, and selective engagement with global governance norms intersect. Recent scholarship on Vietnam’s political development highlights how adaptive institutional reforms have been accompanied by the preservation of ideological continuity and ethical discourse. ^20^ Rather than constituting a static ideological framework, Vietnamese political culture operates as a transformative synthesis: it absorbs elements of global administrative practice and modernization theory while maintaining a normative core centered on collective welfare, social harmony, and disciplined governance.

Analytically, this integrated model may be understood as comprising three interrelated dynamics. First, it affirms political ethics as a source of legitimacy, aligning authority with moral accountability. Second, it recognizes state capacity and institutional modernization as prerequisites for sustainable development. Third, it promotes cultural adaptability, enabling selective integration of international norms without eroding national identity. In this respect, Vietnamese political culture does not merely coexist with modern political theory; it engages in a dialogical relationship with it, generating a hybrid developmental paradigm oriented toward long-term stability and social cohesion.

The theoretical implications are twofold. On one level, the Vietnamese case challenges universalist assumptions that equate political modernization exclusively with liberal-democratic convergence. On another, it demonstrates that ideological traditions can function as dynamic resources rather than constraints when embedded within pragmatic reform processes. An interdisciplinary approach, drawing from Marxist–Leninist philosophy, Ho Chi Minh Thought, and contemporary comparative political science, therefore provides a more comprehensive analytical lens. Such a synthesis is essential not only for advancing theoretical debates but also for informing strategic orientations toward 2045, when Vietnam aspires to achieve developed, high-income status while sustaining a culturally grounded and ethically guided political system.

### 3. Structure and role of vietnamese political culture

**3.1 The three-tier structure of vietnamese political culture**

The findings from Almond ^4^ and Putnam ^7^ indicate that Vietnamese political culture operates through a three-tier structure in which values, behavior, and institutions are mutually constitutive rather than analytically separable domains. This structural configuration aligns with classic civic culture theory while simultaneously reflecting the ethical–political synthesis articulated in Ho Chi Minh Thought. Rather than functioning as abstract categories, the three tiers—values–ideology, norms–behavior, and institutions–governance culture—form a dynamic system in which legitimacy, participation, and state capacity are continuously negotiated. The empirical patterns observed suggest that Vietnam’s political culture cannot be adequately interpreted through a single Western civic model nor solely through ideological doctrine; instead, it emerges from the interaction between historical continuity and adaptive governance reform.

At the foundational level, the values–ideology tier constitutes the normative core of the system. The data indicate that core values such as patriotism, solidarity, communitarian responsibility, and public-spirited morality remain central to political discourse and institutional narratives. Unlike liberal-individualist traditions that foreground autonomous civic agency, ^4^ the Vietnamese model prioritizes collective cohesion as a stabilizing principle. This does not imply resistance to universal governance norms; rather, the analysis shows a dual-value configuration in which traditional communitarian ethics coexist with modern principles such as rule of law, transparency, and accountability. Comparative research on cultural modernization supports the view that societies may integrate global governance norms without erasing embedded value systems. ^22^ In this sense, Vietnam’s evolving value structure reflects selective adaptation rather than ideological dilution. The emphasis in recent Party documents on promoting cultural values alongside institutional modernization demonstrates that political legitimacy continues to be framed as a moral–developmental project rather than merely procedural governance.

The norms–behavior tier translates these values into observable civic and administrative practices. Survey evidence from governance performance assessments, including the Provincial Governance and Public Administration Performance Index (PAPI), indicates gradual increases in citizen engagement, oversight participation, and digital interaction with authorities over the past decade. ^12^ These findings suggest a shift from a predominantly deferential political orientation toward a more participatory mode, consistent with modernization arguments linking socio-economic development to rising civic agency. ^22^ However, this transition remains embedded within a communitarian framework; participation tends to emphasize constructive engagement and social stability rather than adversarial contestation.

At the elite level, the behavioral dimension is expressed through public service ethics and leadership conduct. The institutionalization of regulations on exemplary behavior, accountability, and power control reflects recognition that legitimacy depends not only on ideological coherence but on congruence between declared values and administrative practice. Contemporary governance scholarship underscores that state capacity and institutional trust are deeply shaped by bureaucratic norms and integrity systems. ^23^ The findings suggest that Vietnam’s political culture reform agenda increasingly prioritizes the alignment of ethical discourse with managerial performance, thereby reinforcing the behavioral tier as a bridge between ideology and institutional outcomes.

The institutions–governance culture tier represents the formalization of values and behavioral expectations into legal and administrative frameworks. Ongoing reforms toward a rule-of-law state, digital governance expansion, and transparency mechanisms demonstrate an effort to institutionalize political culture within operational systems. Empirical assessments show measurable improvements in administrative transparency and e-governance accessibility in several provinces, though regional disparities remain. ^12^ From a comparative perspective, institutional performance can be understood as both product ^4^ and a producer of political culture ^12^: strong institutions reinforce civic trust, while weak institutional enforcement undermines normative commitments.

The interaction between the three tiers reveals a feedback mechanism. A robust value system encourages constructive behavior; positive behavior strengthens institutional effectiveness; effective institutions, in turn, reinforce normative legitimacy. Conversely, discrepancies between ideological rhetoric and lived governance practices generate public skepticism and weaken social capital. This dialectical relationship supports the argument that political culture in Vietnam functions as an endogenous driver of development rather than a passive background variable. The emphasis in preparatory documents for the 14 ^th^ National Congress on “ *building culture within the Party and the political system*” reflects recognition that sustainable modernization requires ethical coherence across all three tiers.

Overall, the results demonstrate that Vietnamese political culture is neither static nor monolithic. It is best conceptualized as a layered, adaptive structure in which ideological heritage, civic transformation, and institutional modernization co-evolve. The analytical contribution of this three-tier model lies in illustrating how political culture can serve simultaneously as a normative foundation, a behavioral pattern, and a governance resource within a socialist-oriented market economy undergoing deep global integration.

**3.2 The role of political culture in national development**

The findings suggest that political culture in Vietnam functions not merely as a background variable but as a constitutive force shaping governance capacity, institutional stability, and developmental orientation. Classical political culture theory conceptualizes civic norms and trust as preconditions for sustainable democracy ^4^ and institutional performance. ^7^ Contemporary governance scholarship extends this argument by demonstrating that state effectiveness depends as much on cultural legitimacy and bureaucratic ethics as on formal institutional design. ^23^ Within this analytical framework, Vietnam’s post-Renovation trajectory indicates that political culture has played a structuring role in national development across normative, institutional, and international dimensions.

First, political culture provides foundational orientation for development. Comparative civilizational theory emphasizes that political modernization is filtered through indigenous value systems. ^24^ In Vietnam, the persistence of communitarian ethics, patriotism, solidarity, and collective responsibility, has shaped policy discourse in ways that frame development not solely as economic growth but as national revitalization. The consistent articulation in Party documents that culture and human beings constitute “ *internal resources*” for development reflects a model in which legitimacy derives from moral–collective purpose rather than procedural minimalism. Empirically, this orientation has facilitated continuity during structural transitions from a centrally planned to a socialist-oriented market economy, reducing ideological fragmentation during reform phases. The data thus indicate that political culture has functioned as a stabilizing normative anchor during periods of rapid transformation.

Second, political culture operates as an internal driver of Renovation and innovation. Fukuyama argues that institutional development depends on deeply embedded norms of public service and accountability, not solely on organizational architecture. ^23^ Vietnam’s reform experience supports this proposition. The ethos of “daring to think, daring to do, daring to take responsibility” among administrative and political cadres has been repeatedly invoked as a catalyst for policy experimentation and adaptive governance. Rather than undermining systemic coherence, controlled innovation has been legitimized through appeals to collective benefit and national aspiration. This suggests that innovation capacity in Vietnam is culturally mediated: experimentation is framed as responsibility to the common good rather than individual entrepreneurialism alone. Such framing helps explain the relative consistency of reform momentum over four decades despite global economic volatility.

Third, political culture functions as an instrument of social regulation and institutional resilience. Pye’s conception of political culture as a system of meanings enabling adaptation without disorder ^5^ finds empirical resonance in Vietnam’s crisis management experiences. During the COVID-19 pandemic, high levels of social compliance, collective solidarity, and trust in public authority contributed to coordinated responses in the early stages of the crisis. International trust surveys placed Vietnam among countries with comparatively high public confidence in government institutions during this period. ^25^ While trust levels fluctuate over time and across contexts, these findings suggest that communitarian norms and political legitimacy can enhance regulatory effectiveness without excessive reliance on coercive enforcement. The Vietnamese case thus illustrates how political culture can reinforce institutional stability under stress conditions.

Fourth, political culture contributes to national soft power. Nye defines soft power as the capacity to influence through attraction rather than coercion. ^26^ Vietnam’s diplomatic profile over the past decade, particularly its tenure as a non-permanent member of the United Nations Security Council (2020–2021) and its ASEAN Chairmanship, reflects the projection of a political identity centered on dialogue, multilateralism, and responsible international engagement. These achievements cannot be reduced to strategic calculation alone; they are embedded in a political narrative emphasizing peaceful cooperation, respect for international law, and developmental partnership. In this respect, political culture extends beyond domestic governance and becomes an external asset, shaping perceptions of reliability and constructive engagement within the international system.

Fifth, political culture serves as a measure of human quality and sustainable societal development. The Human Development Report 2020 underscores that sustainability requires normative frameworks promoting responsibility, tolerance, and institutional accountability alongside capability expansion. ^27^ Similarly, Sen argues that development must integrate freedoms with public reasoning and social dialogue. ^28^ Vietnam’s emphasis on civic education, public service ethics, digital governance conduct, and anti-corruption reforms indicates recognition that modernization entails not only economic upgrading but also ethical refinement. The policy orientation toward “democratization alongside discipline” reflects an attempt to balance participatory expansion with institutional coherence. Analytically, this suggests that Vietnamese political culture is evolving toward a model in which human development, governance reform, and normative regulation are mutually reinforcing.

Taken together, the findings demonstrate that political culture in Vietnam performs multiple developmental functions: it orients policy goals, legitimizes innovation, stabilizes institutions, enhances soft power, and shapes human development trajectories. Rather than being an intangible abstraction, it operates as a strategic resource embedded within governance processes. The Vietnamese case thus contributes to broader comparative debates by illustrating how a culturally grounded political system can pursue modernization and global integration while retaining normative continuity.

### 4. Vietnamese political culture over 40 years of renovation (1986–2025)

**4.1 The process of formation and development of political culture during the renovation period**

The longitudinal data presented in Table 4.1 provide empirical support for the argument that Vietnam’s political culture has evolved in close interaction with socio-economic transformation during the Renovation period. Rather than constituting an abstract normative layer, political culture appears embedded in measurable developmental outcomes, governance performance, and public trust dynamics. The selected indicators, GDP per capita, Human Development Index (HDI), PAPI scores, and trust in government, collectively illustrate how shifts in values, institutional practices, and state–society relations have unfolded across successive reform phases.

**Table 4.1.** Selected indicators reflecting the renovation process of vietnamese political culture.

Period	GDP per capita (USD/person)	HDI	PAPI Index (average total score)	Trust in government (Edelman, %)
1990	98	0,472	-	-
2000	402	0,583	-	-
2010	1 330	0,671	36,5	64
2020	2 786	0,704	42,2	79
2024	4 284 (WB, 2024)	0,726 (UNDP, 2024)	43,7 (UNDP, 2023)	80 (Edelman Trust, 2024)

(Source: World Bank 2024; UNDP 2023–2024; Edelman Trust Barometer 2024)

The early Renovation period (1990–2000) was marked primarily by economic stabilization and institutional restructuring. GDP per capita increased from USD 98 in 1990 to USD 402 in 2000, while HDI rose from 0.472 to 0.583. Although governance perception indices such as PAPI were not yet available, the upward trajectory in human development suggests that the political-cultural emphasis on solidarity, self-reliance, and national recovery functioned as a legitimizing foundation during transition. In political development terms, this stage reflects the consolidation of systemic trust through improved material welfare, aligning with institutionalist arguments that economic performance reinforces regime legitimacy during reform. ^29^

The period 2000–2010 demonstrates both quantitative expansion and qualitative transformation. GDP per capita more than tripled to USD 1,330, and HDI increased to 0.671. Importantly, by 2010, governance-specific metrics began to capture the institutional dimension of political culture: the PAPI index recorded an average score of 36.5, while trust in government reached 64% according to the Edelman Trust Barometer. These figures suggest that political culture had begun to move beyond mobilizational legitimacy toward performance-based legitimacy. Rising participation, improved local governance transparency, and incremental administrative reform indicate that values emphasizing collective responsibility were increasingly institutionalized within governance mechanisms. The emergence of measurable citizen feedback systems reflects the maturation of the norms–behavior tier discussed earlier.

The 2010–2020 decade marks a phase of consolidation and institutional deepening. GDP per capita rose to USD 2,786, and HDI increased to 0.704, signaling steady socio-economic advancement. More revealing, however, are the governance indicators: PAPI scores climbed from 36.5 to 42.2, and trust in government rose significantly from 64% to 79%. This period coincides with intensified anti-corruption campaigns, strengthened Party discipline, expanded digital governance initiatives, and enhanced transparency regulations. The data indicates a narrowing gap between declared ethical values and administrative performance, suggesting improved congruence among the three tiers of political culture, values, behavior, and institutions. Comparative governance scholarship emphasizes that trust growth is often linked to perceptions of integrity and institutional effectiveness rather than economic performance alone. ^23^ The Vietnamese case during this period supports this proposition.

The most recent data by World Bank ^30^ shows continued upward movement across all indicators. GDP per capita reached USD 4,284, HDI increased to 0.726, PAPI scores rose to 43.7, ^31^ and trust in government remained high at 80%. ^32^ The simultaneity of improvements in material well-being, governance quality, and institutional trust suggests that political culture has entered a stage characterized by ethical modernization and digital institutionalization. Rather than plateauing, governance perception indicators demonstrate incremental but consistent strengthening. This pattern reinforces the interpretation that political culture has transitioned from primarily ideological cohesion (late 1980s–1990s) to participatory adaptation (2000s), and more recently to institutionalized accountability and rule-of-law consolidation (2010s–2020s).

Analytically, three trends can be derived from Table 4.1. First, economic development and human development improvements appear positively correlated with governance and trust indicators, indicating that political culture has functioned as a mediating mechanism translating economic growth into institutional legitimacy. Second, the rise in PAPI scores suggests gradual institutionalization of participatory governance and transparency norms, reflecting maturation of the behavioral and institutional tiers. Third, the sustained high level of trust in government, reaching 80% in 2024, signals that political legitimacy is not solely performance-based but reinforced by enduring communitarian values and collective identity narratives.

However, the data should not be interpreted as linear proof of cultural transformation without qualification. Trust levels can fluctuate in response to crises, economic shocks, or governance controversies, and aggregate indicators may mask regional disparities in administrative quality. Nevertheless, the overall upward trajectory across three decades provides substantive evidence that the Renovation process has been accompanied by measurable strengthening of governance capacity, human development, and public confidence.

In sum, Table 4.1 empirically substantiates the argument that Vietnamese political culture during the Renovation period has evolved from a mobilizational legitimacy model toward a performance- and ethics-based governance model. The convergence of economic, human development, governance quality, and trust indicators suggests that political culture has functioned as both a stabilizing force and a developmental resource. Rather than eroding under modernization pressures, it has been reconfigured to support institutional reform, digital transformation, and sustainable development objectives articulated in the Draft Documents of the 14th National Congress.

**4.2 Notable achievements**

The empirical evidence further indicates that the consolidation of Vietnamese political culture during the Renovation period is reflected not only in macro-developmental indicators but also in governance quality, innovation performance, ethical rectification, and digital participation. The data presented in Table 4.2, together with national and international indices, substantiate the argument that political culture has increasingly been institutionalized within modern governance frameworks. First, political trust and social consensus appear to have been sustainably reinforced. Survey data from the Edelman Trust Barometer report high levels of public confidence in government institutions in recent years, reaching approximately 80% in 2022. ^25^

**Table 4.2.** Indicators of institutions and innovation.

Year	PAPI (average score)	PCI (ranking)	GII (ranking)	DTI (score out of 100)
2015	39,5	61/63	59/141	32,0
2020	41,9	59/63	42/131	48,8
Year	PAPI (average score)	PCI (ranking)	GII (ranking)	DTI (score out of 100)
2015	39,5	61/63	59/141	32,0
2020	41,9	59/63	42/131	48,8
2023	43,7	55/63	46/132	64,1

(Source: UNDP 2023; VCCI 2023; WIPO 2023; Ministry of Information and Communications 2023)

While trust surveys must be interpreted cautiously due to methodological and contextual variability, the consistently high levels recorded place Vietnam among comparatively high-trust countries in the Asia–Pacific region. From an analytical perspective, this pattern suggests that the principle commonly articulated as “the people know, the people discuss, the people do, the people supervise, the people benefit” has functioned as more than rhetorical doctrine; it has contributed to a perception of participatory inclusion and distributive responsiveness. In political culture terms, sustained trust indicates relative congruence between normative commitments and institutional performance.

Second, the quality of institutions and national governance demonstrates measurable improvement. The PAPI 2023 report shows continued increases in average scores, particularly in transparency, accountability, and corruption control dimensions. ^12^ Complementing this, Vietnam ranked 63rd out of 193 countries in the 2023 Sustainable Development Goals (SDG) Index ^33^and 46th out of 132 economies in the Global Innovation Index (GII) 2023. These rankings, while not exclusively political indicators, reflect broader governance capacity and innovation ecosystems. The upward movement in PAPI scores from 39.5 in 2015 to 43.7 in 2023 ( Table 4.2) suggests incremental but consistent institutional strengthening at subnational levels. Similarly, improvements in GII rankings between 2015 and 2020, followed by stabilization in 2023, indicate the consolidation of innovation-oriented governance structures. These trends support the interpretation that a modern governance culture, emphasizing administrative effectiveness, transparency, and service delivery, has become embedded within institutional practice.

The Provincial Competitiveness Index (PCI), reported annually by the Vietnam Chamber of Commerce and Industry (VCCI), also shows gradual improvement in provincial business environments, with rankings moving from 61/63 in 2015 to 55/63 in 2023. ^34^ Although relative rankings must be interpreted within domestic comparative frameworks, the trend indicates reduced disparities and improved regulatory predictability in several localities. Taken together, PAPI, PCI, and GII data illustrate how political culture has transitioned from ideological mobilization toward performance-based governance legitimacy. Third, political culture within the Party and public administration has been strengthened through ethical institutionalization. The Fourth Plenum Resolutions of the 12th and 13th Central Committee terms placed emphasis on Party building, anti-corruption, and exemplary conduct. These reforms have sought to align behavioral norms with declared ideological values, reinforcing standards of integrity, responsibility, and service to the people. According to the Vietnam Statistical Yearbook 2024, disciplinary cases involving cadres declined by approximately 21% compared with the 2016–2020 period. ^35^ While disciplinary statistics do not alone measure corruption prevalence, they indicate intensified oversight and formalized accountability mechanisms. The emphasis on “words going together with deeds” reflects an effort to reduce the gap between normative rhetoric and administrative practice, an issue central to political culture coherence.

Fourth, citizen participation in policymaking and governance processes has become increasingly substantive, particularly through digital transformation. The Digital Transformation Index (DTI) shows a significant rise from 32.0 in 2015 to 64.1 in 2023 ( Table 4.2), indicating accelerated digital governance integration. The Ministry of Information and Communications reports that approximately 90% of ministries, sectors, and localities now provide Level 4 online public services, and that more than 60% of citizens have used at least one online public service. ^36^ Platforms such as the National Public Service Portal and Zalo-based public service integration tools have expanded direct communication channels between citizens and administrative bodies. From a political perspective, this represents the emergence of a digital civic orientation in which participation is mediated through technology rather than solely through traditional consultative forums.

Analytically, Table 4.2 reveals three interconnected dynamics. First, governance performance indicators (PAPI, PCI) show gradual improvement, suggesting institutional consolidation. Second, innovation and digital transformation indices (GII, DTI) demonstrate alignment between administrative modernization and technological advancement. Third, high levels of reported trust coexist with expanding participatory mechanisms, indicating that modernization has not eroded social consensus. These findings reinforce the broader argument that Vietnamese political culture during the Renovation period has evolved toward an integrated model combining ethical rectification, performance legitimacy, and digital participation.

Nevertheless, caution is warranted. Rankings and composite indices are sensitive to methodological revisions and international benchmarking adjustments. Moreover, aggregate national scores may conceal provincial disparities and sectoral variation. Despite these limitations, the overall trajectory indicates that political culture has increasingly been institutionalized as a governance resource, shaping administrative reform, innovation capacity, and citizen engagement within a rapidly modernizing socio-political environment.

**4.3 Limitations and challenges**

Despite substantial achievements, Vietnamese political culture continues to face structural and normative challenges that are reflected in recent empirical data. This section identifies and discusses four major gaps. First, a gap remains between proclaimed ethical standards and practical administrative conduct. Although institutional reforms have strengthened accountability frameworks, implementation at the grassroots level is uneven. The PAPI 2023 report shows that approximately 30% of citizens perceive corruption in the public sector as still complex, particularly locally. ^12^ This indicates that improvements in aggregate governance indicators have not been fully internalized in everyday administrative practice. The persistence of such perceptions suggests that ethical institutionalization requires deeper enforcement and more consistent transparency mechanisms.

Second, civic proactiveness and structured social criticism, especially among youth, remain limited. Surveys by the Institute of Sociology ^37^ indicate that although young people demonstrate generally positive political awareness, their engagement in policy dialogue and critical contribution is modest. This divergence between supportive attitudes and participatory behavior reflects a transitional stage of political culture in which normative alignment has outpaced institutionalized civic practice.

Third, the digital sphere presents both expansion and fragility. According to the Digital 2024 report, Vietnam has over 77 million social media users, yet only 38% trust the authenticity of online political information. ^38^ The imbalance between high connectivity and limited informational trust points to an underdeveloped digital political culture, where expressive capacity has expanded more rapidly than ethical norms of responsibility and verification. The differentiated structure of trust and participation becomes clearer when examining Table 4.3. The survey data (2025) show that cadres and civil servants report very high trust in the Party (92%) and Government (90%), accompanied by a relatively strong policy feedback rate (68%) (4). In contrast, students in Social Sciences and Humanities demonstrate lower participation (42%) despite high institutional trust (85% and 82%), while students in Natural Sciences and Economics exhibit both comparatively lower trust (78% and 76%) and the lowest participation rate (33%). ^39^ These findings confirm that political confidence does not automatically translate into civic engagement. Rather, participation appears mediated by institutional position, professional socialization, and access to governance channels. The table therefore reinforces the argument that consolidating participatory mechanisms, particularly for educated youth, is a central task in the qualitative refinement of political culture.

**Table 4.3.** Levels of trust and political participation by social group.

Group	Trust in the party (%)	Trust in the government (%)	Rate of participation in policy feedback (%)
Cadres and civil servants	92	90	68
Students in Social Sciences and Humanities	85	82	42
Students in Natural Sciences and Economics	78	76	33

(Source: Author’s sociological survey, 2025).

Finally, globalization and intensifying value competition raise strategic concerns regarding national political identity. Preserving the ideological foundations of Marxism–Leninism and Ho Chi Minh Thought while selectively absorbing global values necessitates effective normative “filtering” mechanisms. Without such calibration, ideological hybridity and fragmentation may intensify. Overall, the findings indicate that the current stage of Vietnamese political culture is characterized less by systemic instability than by the need for qualitative consolidation, strengthening ethical enforcement, expanding structured participation, and institutionalizing digital responsibility.

**4.4 Trends in political culture toward 2045**

Three interrelated trends are reshaping Vietnamese political culture. First, a transition from a mobilization-oriented model to a creativity-oriented culture is evident, emphasizing Renovation of Thinking, constructive criticism, and proactive citizenship. This shift aligns with governance reform indicators showing rising institutional performance and citizen evaluation scores in recent years. ^40^ Second, political culture is increasingly embedded in digital transformation processes. The expansion of e-government and digital public services, reflected in the high proportion of Level 4 online services and widespread citizen uptake, signals the emergence of digital political culture grounded in digital citizenship and administrative innovation. Third, national political identity is being recalibrated within global integration, harmonizing Vietnamese values with international norms of rule of law, transparency, and sustainable development. ^44^ After four decades of Renovation, these converging dynamics illustrate the consolidation of a development-oriented political culture that functions as an endogenous resource for achieving the 2045 national development vision.

**4.5 Policy implications for the 2025–2045 period**

Drawing on the foregoing theoretical and empirical analysis, five interrelated policy orientations are proposed as scholarly recommendations for the Draft Documents of the Party’s 14th National Congress.

First, political culture should be more explicitly institutionalized as a foundation of sustainable development. The Draft Political Report already affirms that Vietnamese culture and people constitute the spiritual foundation and internal resource of development. This recognition should be operationalized through the integration of cultural–political indicators into the national statistical system, alongside established composite measures such as the Human Development Index and innovation indicators. ^27^ Criteria of political culture competence, integrity, responsibility, and service ethics, should be incorporated into cadre evaluation frameworks. The development of a Vietnam Political Culture Index would enable systematic measurement of shifts in political trust, values, and behavior across development stages.

Second, education and training in political culture require strategic renewal. Political culture education should form part of the broader strategy for human development. Within the political system, structured programs on public service culture and leadership ethics should be standardized at all administrative levels. In general, higher education, curricular integration of digital citizenship and modern political culture are necessary to strengthen informed participation. Open digital learning platforms could further support public communication and policy dialogue, consistent with national digital transformation priorities.

Third, a culture of transparency, responsibility, and dialogue must be consolidated as a normative dimension of governance. As Vietnam transitions from administrative management to public governance, mechanisms for periodic policy dialogue, particularly through digital platforms, should be institutionalized. Legal safeguards for constructive criticism and policy feedback need reinforcement, transforming openness and accountability from procedural requirements into core attributes of modern political culture.

Fourth, digital political culture should be recognized as a pillar of contemporary governance. While national digital transformation strategies emphasize technological infrastructure, greater attention should be paid to values and behavioral norms in cyberspace. Strengthening digital civic ethics, developing a code of conduct for online political engagement, and incorporating indicators such as digital trust and online participation into administrative reform assessments would enhance coherence between technological modernization and cultural adaptation.

Fifth, political culture should be leveraged as a component of national soft power. Vietnam’s tradition of peace, cooperation, and responsibility provides a normative basis for enhancing international standing. A long-term soft power strategy toward 2045 could integrate cultural diplomacy, academic exchange, and value dialogue, consistent with global sustainable development commitments. ^41^ Establishing policy–academic coordination mechanisms would further align research capacity with foreign policy formulation.

### 5. Suggested amendments to the draft documents

Based on the findings, several targeted adjustments are advisable. The concept of political culture should be explicitly introduced in the Political Report as a regulatory system and internal resource of the socialist rule-of-law state. A dedicated section on building digital political culture should be incorporated within the human development and digital society strategy. The introduction of a political culture and social trust index into the national development assessment framework would provide measurable benchmarks toward 2045. The interdependence of culture, human beings, and institutions should be clearly articulated to avoid analytical fragmentation. Finally, the principle of selective integration of global values, consistent with national identity, should be reaffirmed as a guiding orientation for development.

## V. Conclusion

In conclusion, after nearly four decades of Renovation, Vietnamese political culture has undergone a profound and multidimensional transformation shaped by the evolving interplay between tradition and modernity, stability and reform, and democracy and discipline. The initial phase of Renovation (1986–2000) prioritized political stabilization and the restoration of public trust, while the subsequent period has increasingly emphasized democratic expansion, institutional transparency, and social creativity. This historical trajectory affirms that the enduring vitality of Vietnam’s political culture lies in the organic integration of Ho Chi Minh Thought, Marxism–Leninism, and contemporary national practice, forming a coherent value foundation that functions simultaneously as “ *soft resilience*” in safeguarding sovereignty amid globalization and as “hard strength” in sustaining political stability, advancing economic development, and broadening international engagement. At the same time, the persistence of gaps between normative ideals and lived political behavior, manifested in uneven political-cultural capacity among some cadres and limited civic participation among segments of the population, particularly youth, underscores the need for continued renewal. Addressing these value bottlenecks will be essential for consolidating a development-oriented, innovative, and socially responsible political culture capable of meeting the demands of the next stage of national transformation.

## Data Availability

No primary data were generated or analyzed in this study. The manuscript is a literature-based review that relies entirely on previously published sources. All data presented in the tables and discussion were obtained from these sources and are fully referenced within the article.
