# VIETNAMESE POLITICAL CULTURE: STRUCTURE, ROLE, AND DEVELOPMENT ORIENTATION IN THE NEW ERA

**DOI:** 10.12688/f1000research.177771.2

**Published:** 2026-08-25

**Authors:** Pham Thi Thuy Van, LAI QUOC KHANH

**Affiliations:** 1 Ha Noi Pedagogical University 2, Phu Tho, Vietnam; 2 Ho Chi Minh National Academy of Politics, Hanoi, Vietnam

**Keywords:** Vietnamese political culture; Political values; Governance culture; Sustainable development; Renovation

## Abstract

This article examines the structure and role of Vietnamese political culture in the context of the country’s new stage of development, considering the orientations set out in the Draft Political Report submitted to the Fourteenth National Congress of the Communist Party of Vietnam. Applying an interdisciplinary approach that integrates Marxist–Leninist theory, Ho Chi Minh Thought, and contemporary political culture studies, the article conceptualizes political culture not only as a spiritual foundation but also as an internal resource, a soft regulatory mechanism, and a driving force for sustainable development. On this basis, the study proposes a threetier analytical framework of Vietnamese political culture, encompassing values and ideology, norms and political behavior, and institutions and governance culture. Drawing on a synthesis of theoretical perspectives and an analysis of Vietnam’s Renovation experience from 1986 to 2025, the article clarifies the dialectical relationship between political culture, institutional quality, governance effectiveness, and social trust. The findings indicate that Vietnamese political culture is undergoing a gradual transformation from a mobilization-oriented model toward a development- and creativity-oriented model, reflecting the interaction between national traditions, socialist ideals, and modern governance principles. The article also outlines policy implications for institutionalizing politicalculture values, strengthening political culture within governance institutions, and supporting Vietnam’s development goals through 2030 and the strategic vision for 2045.

## I. Introduction

Amid globalization, digitalization, and deepening international integration, political culture is increasingly recognized as a fundamental driver of sustainable development. Political culture is not only an element of the political superstructure but also a soft regulative mechanism and a source of influence on political perception, behavior, institutional effectiveness, and state identity. ^1^ Modern development should be analyzed not only based on its growth parameters, but also taking into account such criteria as institutional development, governmental capability, transparency, innovation, and trust, all of which are directly linked to the level of political culture. ^2^ In this sense, political culture constitutes both the normative infrastructure and the adaptive capacity of modern states.

Political culture’s importance in Vietnam has come to the fore especially since the Fourteenth National Congress charted key strategic directions. The major political documents presented at the Congress and accepted by it contain the important recognition that culture and people constitute not just the spiritual pillar of a nation but also a reservoir of internal resources, regulatory mechanisms, and impetus for sustainable development. ^3^ This marks an important shift in political philosophy, as culture is seen to go beyond supporting socio-economic development and becomes a decisive factor influencing it.

The urgency of studying Vietnamese political culture arises from both theoretical and practical considerations. Classical scholarship on political culture, especially the foundational works of Almond and Verba ^4^ and Pye and Verba, ^5^ established the relationship between civic orientations and democratic stability. Subsequent research has further emphasized the role of trust, social capital, ^6^ and institutional effectiveness in sustaining governance systems. ^7^ However, these theoretical models were largely formulated within Western liberal-democratic contexts and may not fully account for the structural and ideological characteristics of socialist-oriented and developing societies, where political culture is closely intertwined with collective value systems and the ruling party’s leadership role. In contrast, Marxism–Leninism and Ho Chi Minh Thought conceptualize politics as inseparable from morality and human development. ^8^ Ho Chi Minh consistently affirmed that political leadership must be grounded in revolutionary ethics and regarded culture as a guiding force illuminating the path of national development. ^9^ Thus, examining Vietnamese political culture today is not only an act of theoretical inheritance but also a necessary effort to develop Marxist–Leninist and Ho Chi Minh thought under contemporary conditions.

From a practical perspective, nearly a century since the founding of the Communist Party of Vietnam and four decades of Renovation ( *Đổi Mới*) have generated profound transformations in Vietnam’s political and social life. ^1^ The country has achieved notable progress in economic growth, innovation performance, ^10^ digital transformation, and international integration. ^11^ At the same time, new challenges have emerged, including concerns regarding public service ethics, ^2^ governance transparency, behavioral norms in digital spaces, and the maintenance of social trust. ^12^ The development of a socialist-oriented market economy and the intensification of global integration, combined with the pressures of the Fourth Industrial Revolution, require a restructuring of political culture along the principles of integrity, accountability, creativity, and civic responsibility. Without a resilient political culture, modernization processes risk generating value dislocation, erosion of trust, and identity fragmentation.

Within global scholarly discourse, ongoing crises of confidence in liberal democratic systems and the rise of populist movements have renewed attention to alternative governance models. ^7^ Some scholars have highlighted the relevance of East Asian political-cultural configurations characterized by strong state capacity, ^6^ communitarian values, and ethical public administration. ^13^ Vietnam’s political culture, shaped by national traditions, socialist ideals, and adaptive integration, offers a distinctive case for comparative analysis in this evolving debate.

Against this backdrop, the present article seeks to systematize the theoretical foundations and analyze the structure and developmental role of Vietnamese political culture in the contemporary period. Drawing on four decades of Renovation practice, the study identifies key dynamics of transformation and proposes orientations toward the 2045 development horizon. Grounded in Marxism–Leninism, Ho Chi Minh Thought, and the Party’s contemporary perspectives on culture and human development, the article conceptualizes political culture as a dynamic and dialectical unity of values, behavioral norms, and institutional structures that collectively generate the nation’s spiritual strength and developmental momentum.

## II. Theoretical overview and ideational foundations

### 1. Western traditions and comparative discourse

The concept of political culture emerged as a central analytical category in comparative political science during the mid-twentieth century. It was systematically articulated in *The Civic Culture* by Almond and Verba, who defined political culture as a pattern of subjective orientations toward political action, encompassing citizens’ attitudes, beliefs, values, and competencies in relation to the political system and their roles within it. ^4^ This formulation marked a significant departure from purely institutional analyses of politics by emphasizing the psychological and normative dimensions underpinning political stability and democratic performance. Expanding this perspective, Pye conceptualized political culture as the total configuration of shared meanings, symbols, and value orientations through which individuals and groups define political roles and legitimate authority. ^5^ In this view, political culture operates not merely as individual disposition but as a collective interpretive framework structuring political life.

Subsequent scholarship diversified the theoretical landscape. Inglehart advanced a modernization-based interpretation, arguing that socio-economic development generates a gradual shift from materialist priorities focused on economic and physical security toward post-materialist values emphasizing autonomy, participation, and self-expression. ^14^ This transformation, he suggested, reshapes political culture and alters the foundations of legitimacy and governance. Building on this line of inquiry, Welzel proposed that emancipative values, centered on individual choice and equal opportunity, constitute an internal cultural force sustaining democratic development and institutional accountability. ^15^ From a complementary institutional perspective, Putnam demonstrated that civic traditions and social capital significantly influence governmental performance, illustrating the reciprocal relationship between cultural norms of trust and the effectiveness of public institutions. ^7^ Together, these approaches converge on the understanding that political culture constitutes a system of collectively shared values and behavioral expectations that mediates the relationship between citizens and the state, thereby shaping governance outcomes.

Despite their analytical sophistication, these theoretical models were largely developed within Western liberal democratic contexts. Their underlying assumption pluralist competition, individualist value systems, and electoral accountability, may not fully capture the structural and ideological configurations of socialist-oriented or developing societies. In such contexts, political culture is closely intertwined with collective value orientations, national traditions, and the guiding role of a ruling party in directing socio-economic transformation. The normative and institutional foundations of political legitimacy may therefore differ substantially from those assumed in liberal-democratic paradigms.

### 2. Marxist-Leninist and Ho Chi Minh thought foundations

Within the Vietnamese context, the ideational foundations of political culture are deeply rooted in Marxism–Leninism and Ho Chi Minh Thought. From a Marxist–Leninist perspective, political culture cannot be understood as a peripheral or purely symbolic phenomenon; rather, it constitutes a dynamic component of the broader superstructural formation that both reflects and shapes material conditions. Economic relations condition the political and ideological superstructure, yet ideology possesses a relative autonomy and transformative capacity once internalized by social actors. ^16^ In this sense, political culture operates dialectically: it emerges from socio-economic relations yet becomes a material force when embedded in collective consciousness and organized political practice.

Lenin’s theoretical development further clarified this mediating function. His conception of political consciousness emphasized the necessity of organized ideological education in transforming spontaneous class awareness into coherent political agency. ^17^ Political culture, in this view, is cultivated through disciplined organization, moral commitment, and collective purpose. The model fuses ethical rigor and institutional discipline, in which legitimacy derives not only from formal authority but also from demonstrable alignment with mass interests and historical mission. ^18^ Consequently, political culture within a Marxist–Leninist framework functions as an integrative mechanism that binds leadership, ideology, and popular mobilization into a coherent political project. Ho Chi Minh’s contributions represent both continuity and contextual innovation within this tradition. Rather than treating culture as subordinate to politics, he conceptualized it as an orienting force shaping the nation’s ethical and developmental trajectory. ^8^ Ho Chi Minh Thought synthesizes Marxist theory with Vietnamese historical traditions and communitarian moral philosophy, producing a distinctive moral-political framework centered on public virtue, collective solidarity, and national independence. ^19^ In this framework, political legitimacy is inseparable from moral character.

Leadership competence is evaluated not solely by administrative efficiency but also by integrity, self-discipline, and devotion to the public good. Political culture thus becomes embodied in ethical conduct and everyday governance practice. Analytically, Ho Chi Minh’s approach reframes political culture as a triadic structure comprising popular orientation, ethical foundation, and openness to civilizational exchange. First, political culture is human-centered, affirming that state power must ultimately serve the people and derive legitimacy from their trust. Second, it is grounded in moral discipline, emphasizing integrity, thrift, impartiality, and responsibility as conditions of sustainable authority. Third, it remains outward-looking and adaptive, capable of integrating global knowledge while preserving national identity. This ethical–institutional synthesis contributes heavily to regime resilience and governance stability during periods of rapid economic transformation. ^20^

Accordingly, the study of Vietnamese political culture requires a theoretical synthesis that moves beyond the dichotomy between Western civic-cultural models and purely institutional analyses. It calls for an approach that integrates value systems, ideological foundations, and governance structures within a dialectical framework. Such an approach recognizes political culture as a dynamic configuration of values, norms, and institutional practices that evolve in response to socio-economic transformation while maintaining continuity with foundational ideological principles.

## III. Research design and methodology

This study adopts a qualitative and interdisciplinary research design to examine the structure and transformation of Vietnamese political culture during the Renovation ( *Đổi Mới*) period from 1986 to 2025. The research is conceptual, interpretive, and analytical in orientation. It does not seek to test causal hypotheses through primary data collection; rather, it develops a theoretically grounded framework for understanding the evolution of political culture in relation to socio-economic transformation and governance reform. The study synthesizes political theory analysis, historical–institutional inquiry, and secondary quantitative interpretation to construct a coherent analytical model of contemporary Vietnamese political culture.

The analytical framework is structured around three interrelated dimensions: foundational ideological orientations, norms and patterns of political behavior, and institutional and governance culture. This tripartite structure functions as a heuristic device for organizing both theoretical reflection and empirical illustration. It enables systematic examination of the interaction between ideological continuity and adaptive governance practices throughout the reform era, while situating Vietnam’s experience within broader debates on political culture and modernization.

The study relies exclusively on documentary analysis and secondary data sources. First, it engages in qualitative content analysis of foundational ideological texts and official documents, including classical Marxist–Leninist works, the writings of Ho Chi Minh, and resolutions and policy documents issued by the Communist Party of Vietnam across successive Party Congresses. These texts are examined to identify recurring conceptual themes, normative principles, and institutional orientations shaping political culture. Second, the research incorporates publicly available secondary statistical indicators and international datasets to contextualize long-term trends in governance performance, human development, innovation capacity, and public trust. These indicators are used descriptively to illustrate structural transformation over time rather than to perform econometric modeling or primary statistical testing.

No primary empirical research involving human participants, such as surveys, interviews, focus groups, or experimental interventions, was conducted for this study. All data used are derived from publicly accessible documents, official publications, and international datasets already in the public domain. Accordingly, the research does not collect personal or identifiable information, does not require informed consent, and is exempt from institutional review board oversight. Methodologically, the research combines qualitative content analysis, historical process tracing, and descriptive interpretation of secondary indicators. Triangulation across ideological texts, institutional documents, and publicly available statistical sources enhances analytical coherence and interpretive robustness. The temporal scope spans from the initiation of *Đổi Mới* reforms in 1986 to 2026, enabling longitudinal assessment of both continuity and transformation in Vietnamese political culture.

## IV. Results and discussion

### 1. An integrated approach: Connecting ideology and modern comparative theory

The comparative analysis of political culture reveals both divergence and convergence between Western civic-cultural theory and the Marxist–Leninist–Ho Chi Minh tradition. Western scholarship has historically emphasized citizens’ beliefs, participatory norms, ^4^ and civic behavior as determinants of democratic stability. ^7^ Within this framework, political culture is often conceptualized as a bottom-up phenomenon that conditions institutional effectiveness through trust, social capital, and value orientations. By contrast, the Vietnamese tradition foregrounds the ethical foundations of political authority and the legitimacy of power as central components of political culture. Rather than focusing primarily on individual attitudes, this tradition conceptualizes political culture as a morally structured relationship between leadership and the people, mediated by ideological coherence and collective purpose.

Despite these differences in emphasis, contemporary comparative research suggests that rigid dichotomies between Western and non-Western models are increasingly untenable. Fukuyama’s analysis of political order argues that sustainable development depends on the interplay between state capacity, rule-based governance, and cultural norms that reinforce institutional discipline and public trust. ^6^ His assessment of East Asian developmental trajectories underscores the importance of communitarian values and administrative integrity, traits that resonate with elements of Marxist–Leninist political ethics. Similarly, Inglehart and Welzel’s revised modernization theory acknowledges that governance quality is shaped by culturally embedded value systems and need not replicate Western historical pathways, provided governance institutions maintain effectiveness and public legitimacy. ^21^ Political cultures, therefore, emerges as a crucial mediating variable across diverse developmental models, linking normative orientations with institutional outcomes.

Situated within this evolving theoretical landscape, Vietnam represents a distinctive case in which Marxist–Leninist principles, Ho Chi Minh’s moral–political philosophy, and selective engagement with global governance norms intersect. Modern institutional reforms have been accompanied by the preservation of ideological continuity and ethical discourse. ^20^ Rather than constituting a static framework, Vietnamese political culture operates as a transformative synthesis: it absorbs elements of global administrative practice and modernization theory while maintaining a normative core centered on collective welfare, social harmony, and disciplined governance.

### 2. Structure and role of Vietnamese political culture

**2.1 The three-tier structure of Vietnamese political culture**

**Figure 1.**
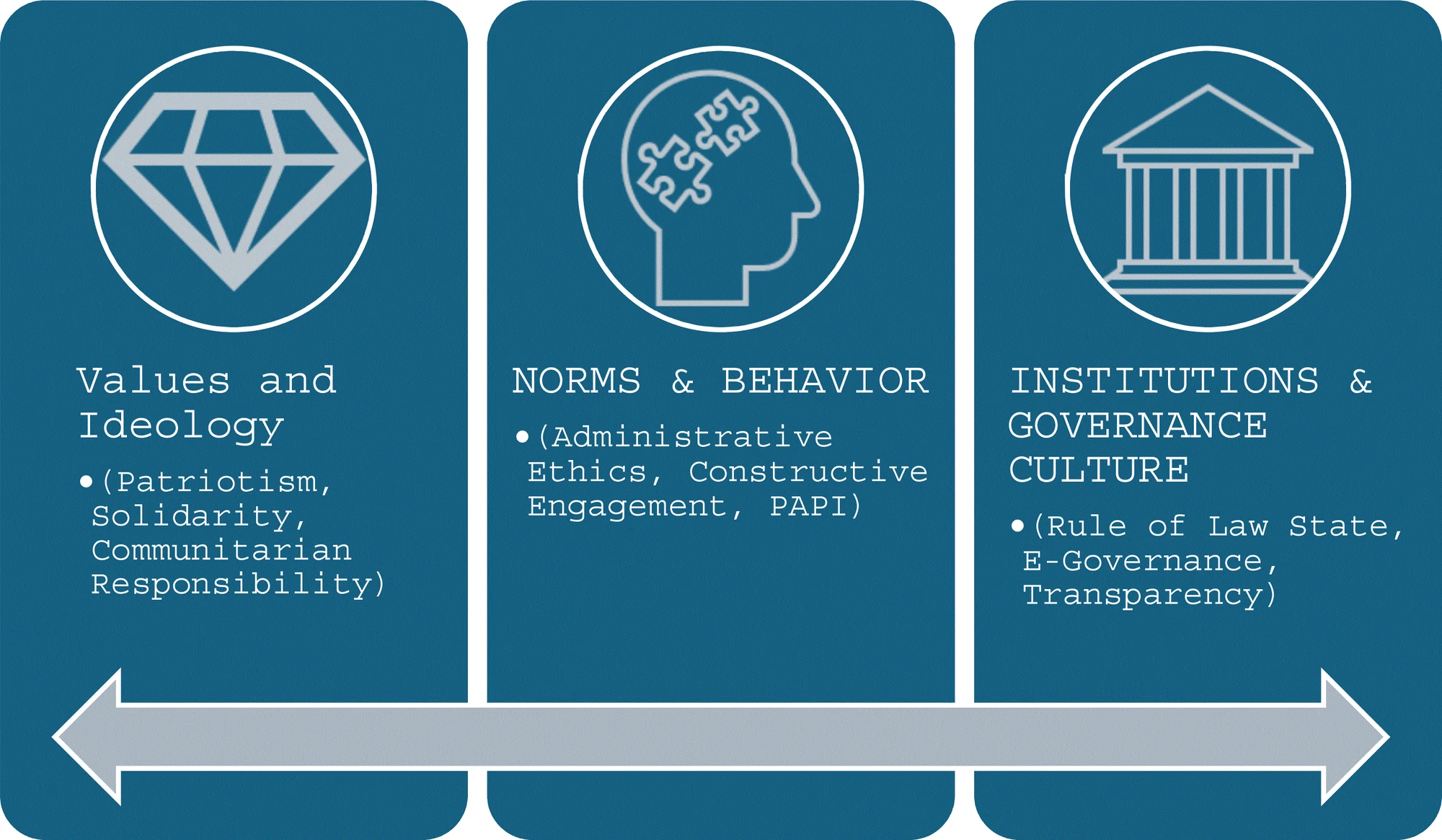
Three-Tier Structure of Vietnamese Political Culture.

Reflecting classic comparative definitions put forth by Almond ^4^ and Putnam, ^7^ alongside domestic tenets, Vietnamese political culture operates through a three-tier structure in which values, behavior, and institutions are mutually constitutive rather than analytically separable domains. Rather than functioning as abstract categories, the three tiers, values–ideology, norms–behavior, and institutions–governance culture, form a dynamic system in which legitimacy, participation, and state capacity are continuously negotiated as represented in Figure 1.

The first tier, labeled The Values-Ideology Tier, constitutes the normative core of the system. Core values such as patriotism, solidarity, communitarian responsibility, and public-spirited morality remain central to political discourse and institutional narratives. Unlike liberal-individualist traditions that foreground autonomous civic agency, the Vietnamese model prioritizes collective cohesion as a stabilizing principle. This does not imply resistance to universal governance norms; rather, the analysis shows a dual-value configuration in which traditional communitarian ethics coexist with modern principles such as the rule of law, transparency, and accountability. ^22^

The second tier, labeled The Norms–Behavior Tier, translates core values into observable civic and administrative practices. Secondary evidence from governance performance assessments, including the Provincial Governance and Public Administration Performance Index (PAPI), indicates gradual increases in citizen engagement, local oversight participation, and digital interaction with authorities over the past decade. ^12^ This transition remains embedded within a cooperative framework; participation tends to emphasize constructive engagement and social stability rather than adversarial contestation. ^22^ At the elite level, the behavioral dimension is expressed through public service ethics, accountability, and power control, prioritizing the alignment of ethical discourse with managerial performance. ^23^ To ensure empirical rigor, clear boundaries must be maintained between individual bureaucratic behavior and the broader institutional design that dictates it.

The tier of The Institutions–Governance Culture represents the formalization of values and behavioral expectations into legal and administrative frameworks. Ongoing reforms toward a socialist rule-of-law state, the expansion of digital governance, and transparency mechanisms demonstrate efforts to institutionalize political culture within operational systems. ^12^ Strong institutions reinforce civic trust, while weak institutional enforcement can undermine normative commitments. Overall, the interaction between these three tiers reveals a critical feedback mechanism: a robust value system encourages constructive behavior; positive behavior strengthens institutional effectiveness; effective institutions, in turn, reinforce normative legitimacy.

**2.2 The role of political culture in national development**

The findings suggest that political culture in Vietnam functions as a constitutive force shaping governance capacity, institutional stability, and developmental orientation. ^7^ Within this analytical framework, Vietnam’s post-Renovation trajectory indicates that political culture has played a structuring role in national development across several key dimensions.

First, political culture provides a foundational orientation for development. In Vietnam, the persistence of communitarian ethics, patriotism, and solidarity has shaped policy discourse, framing development not solely as economic growth but as national revitalization. ^23^ This orientation has facilitated continuity during structural transitions from a centrally planned to a socialist-oriented market economy, reducing ideological fragmentation during reform phases. ^24^ Thus, political culture functions as a stabilizing normative anchor during periods of rapid transformation.

Second, political culture operates as an internal driver of Renovation and innovation. The ethos of “ *daring to think, daring to do, daring to take responsibility*” among administrative and political cadres has been repeatedly invoked as a catalyst for policy experimentation and adaptive governance. ^23^ Rather than undermining systemic coherence, controlled innovation has been legitimized through appeals to collective benefit and national aspiration, helping explain the relative consistency of reform momentum over four decades.

Third, political culture serves as an instrument of social regulation and institutional resilience. During major public crises, such as the early stages of the COVID-19 pandemic, high levels of social compliance, collective solidarity, and trust in public authority contributed to coordinated domestic responses. While trust levels fluctuate across contexts, these patterns suggest that communitarian norms can enhance regulatory effectiveness, though they must be understood alongside concrete administrative enforcement, legal sanctions, and public health awareness. ^25^

Fourth, political culture contributes to national soft power. Vietnam’s diplomatic profile over the past decade, particularly its tenure as a non-permanent member of the United Nations Security Council and its ASEAN Chairmanship, reflects a political identity centered on dialogue, multilateralism, and responsible international engagement. ^26^ Fifth, political culture serves as a measure of human quality and sustainable societal development. The policy orientation toward balancing participatory expansion with institutional coherence indicates recognition that modernization entails not only economic upgrading but also ethical refinement, public service ethics, and comprehensive anti-corruption reforms.

### 3. Vietnamese political culture over 40 years of renovation (1986–2025)

**3.1 The process of formation and development**

The longitudinal data presented in Table 1 provide empirical support for the argument that Vietnam’s political culture has evolved in close interaction with socio-economic transformation during the Renovation period. Macro-developmental indicators, human development scores, and public trust dynamics collectively illustrate how shifts in values, institutional practices, and state–society relations have unfolded across successive reform phases. ^27^ The early Renovation period (1986–2000) focused primarily on economic stabilization and institutional restructuring. GDP per capita increased from USD 98 in 1990 to USD 402 in 2000, while the Human Development Index (HDI) rose from 0.472 to 0.583. During this transition, the political-cultural emphasis on solidarity and national recovery functioned as a legitimizing foundation, consolidating systemic trust through improved material welfare. ^28^

**Table 1.** Selected indicators reflecting the renovation process of vietnamese political culture.

Year	GDP per capita (USD)	HDI	PAPI Index (average total score)	Trust in government (Edelman, %)
1990	98	0.472	-	-
2000	402	0.583	-	-
2010	1 330	0.671	36.5	64
2020	2 786	0.704	42.2	79
2024	4 284	0.726	43.7	80

Source: World Bank (2024), UNDP (2023–2024), Edelman Trust Barometer (2024).

The period 2000–2010 demonstrated both quantitative expansion and qualitative transformation. GDP per capita rose to USD 1,330, and HDI increased to 0.671. By 2010, governance-specific metrics began to capture the institutional dimension of political culture: the PAPI index recorded an average score of 36.5, while trust in government reached 64% according to the Edelman Trust Barometer. This phase reflects a gradual shift toward performance-based legitimacy, in which rising participation and incremental administrative reforms institutionalized collective responsibility. The subsequent decade (2010–2020) marked a phase of consolidation and institutional deepening. GDP per capita rose to USD 2,786, and HDI increased to 0.704. Governance indicators climbed notably, with PAPI scores reaching 42.2 and trust in government rising to 79%. ^23^ This period coincided with intensified anti-corruption campaigns, strengthened Party discipline, and expanded digital governance initiatives, suggesting improved congruence among the three tiers of political culture.

The most recent consolidated datasets from the World Bank ^29^ demonstrate continued upward movement across these dimensions. GDP per capita reached USD 4,284, HDI increased to 0.726, PAPI scores rose to 43.7, ^30^ and trust in government remained resilient at 80%. ^31^ While macro indicators like GDP, HDI, and digital indices are valuable, they should be interpreted as indirect correlates rather than absolute, direct measures of internal political-cultural shifts. Nonetheless, the overall upward trajectory over three decades indicates that political culture has successfully transitioned from primarily historical-ideological cohesion toward institutionalized accountability and rule-of-law consolidation. ^23^

**3.2 Notable achievements**

The macro-developmental indicators are reinforced by improvements in governance quality, innovation performance, ethical rectification, and digital participation. The data presented in Table 2 substantiate the argument that political culture has increasingly been integrated within modern governance frameworks. First, political trust and social consensus appear to have been sustainably reinforced. Consistently high levels of public confidence place Vietnam among comparatively high-trust nations in the Asia–Pacific region. From an analytical perspective, this pattern suggests that the foundational governance principles regarding popular feedback and oversight have contributed to a perception of participatory inclusion and distributive responsiveness. Second, the structural quality of institutions and national governance demonstrates measurable improvement. ^25^ Vietnam ranked 63rd out of 193 countries in the Sustainable Development Goals (SDG) Index ^32^ and 46th out of 132 economies in the Global Innovation Index (GII). ^33^ The upward movement in PAPI scores from 39.5 in 2015 to 43.7 in recent assessments suggests incremental but consistent institutional strengthening at subnational levels. Similarly, improvements in the Provincial Competitiveness Index (PCI), reported by the Vietnam Chamber of Commerce and Industry (VCCI), indicate a more predictable regulatory environment across diverse localities. ^34^

**Table 2.** Key institutional, competitiveness, and innovation indicators.

Year	PAPI (average score)	PCI (ranking)	GII (ranking)	DTI (score out of 100)
2015	39.5	61/63	59/141	32.0
2020	41.9	59/63	42/131	48.8
2023	43.7	55/63	46/132	64.1

Source: UNDP (2023), VCCI (2023). WIPO (2023), Ministry of Information and Communications (2023).

Third, political culture within the public administration has been strengthened through ethical institutionalization. Central Committee resolutions have placed heavy emphasis on Party building, anti-corruption, and exemplary conduct, aiming to align behavioral norms with declared values and reduce the gap between normative rhetoric and administrative practice. ^35^ Fourth, citizen participation in governance processes has become increasingly substantive through digital transformation. The Digital Transformation Index (DTI) shows a significant rise from 32.0 in 2015 to 64.1 in recent years, indicating accelerated integration of digital governance. ^36^ Platforms such as the National Public Service Portal have expanded direct communication channels, encouraging the emergence of an adaptive digital civic orientation.

**3.3 Limitations and challenges**

Despite substantial achievements, Vietnamese political culture continues to face structural and normative challenges. First, an operational gap remains between proclaimed ethical standards and practical administrative conduct at certain grassroots levels. Governance reports show that a notable portion of citizens still perceive local public-sector transparency as complex, indicating that improvements in aggregate indices are unevenly distributed and require stronger enforcement. ^12^ Second, structured social criticism and active civic proactiveness, particularly among certain youth cohorts, remain in a transitional stage. Sociological data indicate that while young people demonstrate highly supportive political awareness, their consistent, structured engagement in formal policy dialogue can be further enhanced. This divergence underscores that normative alignment sometimes outpaces institutionalized civic practice. ^37^

Third, the rapid expansion of the digital sphere poses vulnerabilities to informational trust. While Vietnam has tens of millions of active social media users, a substantial portion express concern about the authenticity of online political information. ^38^ This imbalance points to an ongoing need to cultivate digital citizenship norms, emphasizing responsibility, digital literacy, and information verification alongside pure connectivity. ^39^ Finally, globalization and intensifying global value competition raise strategic concerns regarding national political identity. Preserving the foundational tenets of Marxism–Leninism and Ho Chi Minh Thought while selectively absorbing global administrative practices necessitates effective normative filtering mechanisms. The current stage of Vietnamese political culture is thus characterized less by systemic instability than by the need for qualitative consolidation: strengthening ethical enforcement, expanding structured avenues for feedback, and institutionalizing digital responsibility.

**3.4 Trends in political culture toward 2045**

Three interrelated trends are reshaping the domestic political-cultural landscape. First, a transition from a mobilization-oriented model to a creativity-oriented culture is evident, emphasizing the renovation of thinking, constructive feedback, and proactive citizenship. ^40^ Second, political culture is increasingly embedded in digital transformation processes, signaling the emergence of digital citizenship norms grounded in administrative innovation. ^41^ Third, national political identity is being carefully recalibrated within global integration, harmonizing traditional Vietnamese values with modern international principles of transparency and sustainable development. ^35^

**3.5 Policy implications for the 2025–2045 period**

Drawing on the foregoing theoretical and documentary analysis, five interrelated policy orientations are proposed to guide Vietnam’s developmental trajectory toward the 2045 horizon. First, there is a critical need to operationalize cultural–political awareness by integrating qualitative governance indicators into national statistical frameworks alongside established composite measures like the Human Development Index (HDI). ^42^ Within this framework, criteria of integrity, responsibility, and service ethics must remain central to cadre evaluation systems. Second, modernizing political culture education within both the public administration system and general higher education is essential; this can be achieved by incorporating dedicated modules on digital citizenship, administrative ethics, and constructive dialogue to cultivate more informed civic participation. ^41^ Third, mechanisms for periodic policy dialogue should be institutionalized, particularly through accessible digital platforms, thereby converting procedural openness and administrative accountability into core, everyday attributes of modern governance. Fourth, policymakers must formulate robust codes of conduct for online political engagement, intentionally incorporating digital trust, data privacy, and online responsibility into ongoing administrative reform assessments. Lastly, it is vital to integrate Vietnam’s historical traditions of cooperation, ^41^ multilateralism, and diplomatic dialogue into a comprehensive, long-term international soft power strategy that aligns domestic research capacity with global sustainable development commitments. ^43^

**3.6 Suggested amendments to the draft documents**

Based on the analytical findings, several targeted adjustments are advisable for ongoing strategic planning. The explicit concept of political culture should be systematically utilized as an internal resource of the socialist rule-of-law state. A dedicated subsection on building digital political culture should be robustly incorporated within human development and digital society strategies. Furthermore, the close interdependence among culture, human beings, and institutions should be clearly articulated across policy sectors to avoid analytical fragmentation and to reaffirm the selective integration of global values consistent with national identity.

## V. Conclusion

In conclusion, after four decades of Renovation, Vietnamese political culture has undergone a profound and multidimensional transformation shaped by the evolving interplay between tradition and modernity, stability and reform, and democracy and discipline. The initial phase of Renovation (1986–2000) prioritized political stabilization and the restoration of public trust, while the subsequent period has increasingly emphasized administrative expansion, institutional transparency, and social creativity. This historical trajectory affirms that the enduring vitality of Vietnam’s political culture lies in the organic integration of Ho Chi Minh Thought, Marxism–Leninism, and contemporary national practice, forming a coherent value foundation that functions simultaneously as “ *soft resilience*” in safeguarding sovereignty amid globalization and as “ *hard strength*” in sustaining political stability and advancing economic development. At the same time, the persistence of gaps between normative ideals and practical political behavior highlights the need for continued renewal. Addressing these remaining value bottlenecks will be essential for consolidating a development-oriented, innovative, and socially responsible political culture capable of meeting the demands of the next stage of national transformation toward 2045.

## Data Availability

No primary data were generated or analyzed in this study. The manuscript is a literature-based review that relies entirely on previously published sources. All data presented in the tables and discussion were obtained from these sources and are fully referenced within the article.
