# Supplementary material for: VIETNAMESE POLITICAL CULTURE: STRUCTURE, ROLE, AND DEVELOPMENT ORIENTATION IN THE NEW ERA
Source: F1000Res. 2026 Aug 25;15:676. Originally published 2026 May 7. [Version 2] doi: 10.12688/f1000research.177771.2 (PMC13562953; doi:10.12688/f1000research.177771.2)
Supplement: Peer review history

# Reviewer report: Reviewer response for version 2

**DOI:** 10.5256/f1000research.205037.r514356

**Published:** 2026-09-07

**Authors:** Nguyen Duy Quynh

**Affiliations:** 1 VNU University of Social Sciences and Humanities, Hanoi, Hanoi, Vietnam

I have reviewed the revised manuscript and the authors’ responses to my previous comments. I confirm that the revisions have adequately addressed my concerns, and the manuscript now meets the requirements raised in my previous review.

 Therefore, I am happy to change my previous status from “Approved with reservations” to “Approved”.

Is the work clearly and accurately presented and does it cite the current literature?

Yes

If applicable, is the statistical analysis and its interpretation appropriate?

Yes

Are all the source data underlying the results available to ensure full reproducibility?

Partly

Is the study design appropriate and is the work technically sound?

Partly

Are the conclusions drawn adequately supported by the results?

Yes

Are sufficient details of methods and analysis provided to allow replication by others?

Partly

Reviewer Expertise:

Asia Pacific political culture

I confirm that I have read this submission and believe that I have an appropriate level of expertise to confirm that it is of an acceptable scientific standard.

# Reviewer report: Reviewer response for version 1

**DOI:** 10.5256/f1000research.196055.r485093

**Published:** 2026-05-29

**Authors:** Nguyen Duy Quynh

**Affiliations:** 1 VNU University of Social Sciences and Humanities, Hanoi, Hanoi, Vietnam

**Article title: Vietnamese political culture: structure, role, and development orientation in the new era**

**General assessment**

 In general, this manuscript addresses a significant, timely, and intellectually important subject: the structure, role, and future orientations of Vietnamese political culture within the framework of national development, institutional reform, digital transformation, and Vietnam's strategic vision toward 2045. The authors are to be commended for undertaking an ambitious and interdisciplinary study that seeks to connect Marxist–Leninist theory, Ho Chi Minh Thought, and contemporary political culture studies. This is a valuable and necessary line of inquiry, especially for scholars interested in non-Western pathways to political development, governance legitimacy, state capacity, and development-oriented political systems.

 A notable strength of the manuscript lies in its effort to move beyond a narrow understanding of political culture as merely symbolic, ideological, or rhetorical. Instead, the authors seek to conceptualize Vietnamese political culture as a potential endogenous resource for sustainable development, ethical administration, institutional modernization, social trust, and national soft power. This is a promising and intellectually productive direction. The proposed three-tier framework—values and ideology; norms and political behavior; institutions and governance culture—also has considerable analytical potential. If more clearly defined and operationalized, it could provide a useful framework for examining the relationships among ideology, political behavior, institutional performance, and governance culture in Vietnam.

 The manuscript is therefore a worthwhile contribution in terms of topic selection, theoretical ambition, and policy relevance. It brings together important debates on political culture, governance reform, and Vietnam's long-term development orientation. At the same time, several substantive issues need to be addressed before the article can be assessed as methodologically and analytically satisfactory. These issues do not diminish the value of the authors' broader scholarly effort; rather, they indicate that the manuscript requires further refinement to meet academic standards of methodological transparency, conceptual clarity, and evidentiary support. The main concerns relate to the stated research design, the use of author-generated survey data, the insufficient specification of the qualitative method, the overinterpretation of descriptive indicators, unclear conceptual boundaries within the three-tier framework, and inconsistencies in data presentation.

 With substantial revision, this manuscript has the potential to make a meaningful contribution to scholarship on Vietnamese political culture, governance reform, and non-Western approaches to political development.

**The main comments are:**

***1. Methodological contradiction regarding primary survey data***

 There is an important point for clarification regarding the internal consistency of the research design. In the methodology section, the authors state that the study relies entirely on documentary analysis and secondary data, and that no primary empirical investigation involving human participants (including surveys, interviews, focus groups, or experiments) was conducted. On that basis, the manuscript also states that ethical approval or informed consent was not required.

 However, Section 4.3 includes Table 4.3 and explicitly identifies "Author's sociological survey, 2025" as the source of the data. The surrounding discussion also cites "survey data (2025)," which directly contradicts the authors' claim that no survey was conducted and the evidence in Table 4.3. The authors should therefore revise the methodology to reflect accurately whether such a survey was conducted. If no sociological survey was conducted, Table 4.3 and all related analyses should be removed.

 Resolution of this issue should be considered a high priority. If the survey data are retained, the authors need to provide comprehensive methodological information, including sample size, sampling strategy, recruitment methods, respondent profile, survey questionnaire design, survey location, fieldwork dates, consent procedures, ethical approval or exemption, methods for processing and analyzing the collected data, and access to anonymized data where appropriate. Without this information, Table 4.3 cannot be reproduced and should not be treated as empirical evidence.

***2. The authors overinterpret descriptive indicators as evidence of changes in political culture.***

 The authors provide the following as evidence that Vietnamese political culture has changed over time: GDP per capita, HDI, PAPI, PCI, GII, DTI, and trust survey data. While these indicators are important and informative in the context of Vietnam's development trajectory, none is a definitive indicator of political culture.

 The authors at times move from descriptive indicators to broader claims about change in Vietnamese political culture. For instance, increases in GDP per capita or digital transformation scores may suggest economic development or modernization of the administrative system. Still, they cannot directly demonstrate changes in political values, behavior, civic trust, or governance culture in Vietnam. Similarly, while PAPI and trust survey data can shed light on individuals' perceptions of governance, the authors do not explicitly articulate how these data operationalize the broader concept of political culture.

 To bridge this analytical gap, I highly recommend recalibrating the conclusions drawn from these datasets. The manuscript would be significantly improved if the authors explicitly framed these indicators as indirect, contextual evidence of political-cultural evolution, rather than treating them as definitive proof.

***3. Caution is needed when making causal claims.***

 Numerous statements in the manuscript imply causal relationships between political culture and governance outcomes, social trust, innovation, pandemic compliance, or soft power. While these are reasonable and theoretically compelling hypotheses, the manuscript does not yet convincingly establish these causal links. The authors present an overview of public compliance with measures to curb COVID-19 and argue that social compliance in Vietnam demonstrates the influence of political culture as a form of social regulation. While this may be partially true in the case of Vietnam's long-held tradition of communal responsibility and the state's ability to mobilize society, other factors may also have influenced compliance with COVID-19 measures, such as fear of COVID-19, public health messaging, administrative enforcement, legal sanctions against non-compliance, and local monitoring mechanisms.

 The authors should therefore present political culture as one factor among many that may have influenced compliance with COVID-19 measures, rather than as the sole or primary influence. The same caution should be applied to the relationships among political culture, innovation, digital governance, social trust, and international soft power.

***4. Corrections and standardization are needed for the tables***

 While the tables contain useful information, several require considerable revision. For example, Table 4.2 contains repeated rows and repeated headings. Table 4.1 should be checked for consistency in units of measurement, years of measurement, and numerical presentation. Furthermore, the timeline structure between Table 4.1 and Table 4.2 (e.g., using the milestones 2010, 2015, 2020, 2023) is currently disjointed and lacks logical consistency, making it difficult for the reader to cross-reference the data accurately.

***5. Alignment with official documents of the 14th National Congress of the Vietnamese Communist Party***

 The manuscript appears to have been written in a pre-Congress context, as it refers to the Draft Political Report submitted to the Fourteenth National Congress and offers recommendations for the draft documents. It is acceptable, but the authors should clarify this temporal and documentary context in the introduction or methodology section. Since the official Congress Documents have now been published, the authors may either retain the article as a pre-Congress scholarly contribution with a brief explanatory note or update the manuscript by engaging directly with the official ***documents of the 14th National Congress of the Vietnamese Communist Party***. The latter option would strengthen the article's policy relevance by discussing how the Party's orientations on culture, political culture, governance, and human development can be institutionalized in the coming period. In either case, the distinction between the empirical period of 1986–2025 and the updated policy-document context should be made clear.

Is the work clearly and accurately presented and does it cite the current literature?

Yes

If applicable, is the statistical analysis and its interpretation appropriate?

Yes

Are all the source data underlying the results available to ensure full reproducibility?

Partly

Is the study design appropriate and is the work technically sound?

Partly

Are the conclusions drawn adequately supported by the results?

Yes

Are sufficient details of methods and analysis provided to allow replication by others?

Partly

Reviewer Expertise:

Political culture and Vietnamese politics.

I confirm that I have read this submission and believe that I have an appropriate level of expertise to confirm that it is of an acceptable scientific standard, however I have significant reservations, as outlined above.

# Response

**Published:** 2026-06-27

**Authors:** QUOC KHANH LAI

**Affiliations:** Not Applicable, Vietnam

**The main comments are:**

***1. Methodological contradiction regarding primary survey data***

 It is a major concern regarding the internal consistency of the research design. In the methodology section, the authors state that the study relies entirely on documentary analysis and secondary data, and that no primary empirical investigation involving human participants (including surveys, interviews, focus groups, or experiments) was conducted. On that basis, the manuscript also states that ethical approval or informed consent was not required.

 However, Section 4.3 includes Table 4.3 and explicitly identifies "Author's sociological survey, 2025" as the source of the data. The surrounding discussion also cites "survey data (2025)," which directly contradicts the authors' claim that no survey was conducted and the evidence in Table 4.3. The authors should therefore revise the methodology to reflect accurately whether such a survey was conducted. If no sociological survey was conducted, Table 4.3 and all related analyses should be removed.

 Resolution of this issue should be considered a high priority. If the survey data are retained, the authors need to provide comprehensive methodological information, including sample size, sampling strategy, recruitment methods, respondent profile, survey questionnaire design, survey location, fieldwork dates, consent procedures, ethical approval or exemption, methods for processing and analyzing the collected data, and access to anonymized data where appropriate. Without this information, Table 4.3 cannot be reproduced and should not be treated as empirical evidence.

***The manuscript has been revised accordingly. The tables have references from secondary sources. Survey data relating to the author has been removed.***

***2. Limitations in qualitative methodology and documentary analysis***

 The authors describe their methodology as qualitative content analysis, historical–institutional inquiry, process tracing, and descriptive interpretation of secondary indicators; however, the methodological explanation remains too general.

 No explanation is provided for how the documents were selected or for the inclusion and exclusion criteria applied. A clearer account of coding procedures for this conceptual and interpretive work would help readers understand how recurring themes were identified in Party documents, Ho Chi Minh's writings, Marxist–Leninist literature, and a wide range of national and international data sources. In addition, more details about how the methodology applies to ideological materials, bureaucratic practices, and quantitative indicators would help readers understand how the results could be verified or analyzed by other researchers.

***The methodology section has been revised accordingly.***

***3. While the three-part model is promising, it remains a work in progress.***

 The three-part framework of Vietnamese political culture represents one of the most significant contributions of this manuscript. However, the three tiers of political culture would benefit from greater specificity regarding the differences between layers.

 The first tier (values and ideology) is relatively easy to define. It would include elements such as patriotism and national unity, shared values, collective responsibility, public service ethics, and the principles of Ho Chi Minh's moral-political philosophy. The second and third tiers, however, need clearer differentiation, especially where norms, political behavior, administrative ethics, accountability, transparency, and governance culture overlap. Specifically, the authors are requested to provide a more convincing structural system for clearly defining the boundaries between "norms and political behavior clearly" (the second tier) and "institutions and governance culture" (the third tier). If the authors offered a clearer definition of their theoretical framework, they would be less likely to confuse indicators of good governance in general with indicators of change in Vietnam's political culture specifically.

***The section has been revised to accordingly by explicitly defining the boundary between Tier 2 and Tier 3, as well as separating behavioral manifestations from institutional structures.***

***4. The authors overinterpret descriptive indicators as evidence of changes in political culture.***

 The authors provide the following as evidence that Vietnamese political culture has changed over time: GDP per capita, HDI, PAPI, PCI, GII, DTI, and trust survey data. While these indicators are important and informative in the context of Vietnam's development trajectory, none is a definitive indicator of political culture.

 The authors at times move from descriptive indicators to broader claims about change in Vietnamese political culture. For instance, increases in GDP per capita or digital transformation scores may suggest economic development or modernization of the administrative system. Still, they cannot directly demonstrate changes in political values, behavior, civic trust, or governance culture in Vietnam. Similarly, while PAPI and trust survey data can shed light on individuals' perceptions of governance in Vietnam, the authors do not articulate how these data operationalize political culture.

 The authors need either to improve their operationalization of political culture or to limit their conclusions. The manuscript would be improved if the authors indicated that these indicators provide indirect evidence of change in political culture rather than treating them as definitive evidence.

***The section has been revised accordingly by repeatedly clarifying that the indicators are contextual, indirect, or illustrative, not direct operationalizations of political culture.***

***5. Caution is needed when making causal claims.***

 Numerous statements in the manuscript imply causal relationships between political culture and governance outcomes, social trust, innovation, pandemic compliance, or soft power. While these are reasonable and theoretically compelling hypotheses, the manuscript does not yet convincingly establish these causal links. The authors present an overview of public compliance with measures to curb COVID-19 and argue that social compliance in Vietnam demonstrates the influence of political culture as a form of social regulation. While this may be partially true in the case of Vietnam's long-held tradition of communal responsibility and the state's ability to mobilize society, other factors may also have influenced compliance with COVID-19 measures, such as fear of COVID-19, public health messaging, administrative enforcement, legal sanctions against non-compliance, and local monitoring mechanisms.

 The authors should therefore present political culture as one factor among many that may have influenced compliance with COVID-19 measures, rather than as the sole or primary influence. The same caution should be applied to the relationships among political culture, innovation, digital governance, social trust, and international soft power.

***The section has been revised accordingly.***

***6. The results and discussion section should also be modified.***

 The results and discussion section contains a large amount of theoretical material, particularly regarding Marxism–Leninism, Ho Chi Minh Thought, and the integration of Western political culture theory into the Vietnamese ideological tradition, much of which is redundant with the existing theoretical overview.

 A clearer delineation between the theoretical foundation, methodology, findings, and discussion would improve the paper. The authors should move most of the Marxist–Leninist and Ho Chi Minh sections to the theoretical framework. The results section should focus on what the authors found through document analysis and secondary indicators. This change would improve the manuscript's coherence and reduce redundancy.

***The section has been revised accordingly by limiting the results and discussion section to the presentation and the discussion of new findings.***

***7. The authors' data availability statement also needs to be updated.***

 The current data availability statement says that no primary data were produced or evaluated and that all data were based on previously published works. The "Author's sociological survey, 2025" is incompatible with this statement.

 Depending on the authors' final determination regarding the survey, the following revisions should be made. If the survey remains in place, the authors should make anonymized data and supporting documents available, subject to ethical constraints. If the survey is not retained, the data availability statement should specify the sources for each table, where possible, with stable URLs, report titles, page numbers, and access dates.

***The manuscript used secondary data as stated in the methodology section. Hence the Author’s sociological survey, 2025 has been removed.***

***8. Corrections and standardization are needed for the tables***

 While the tables contain useful information, several require considerable revision. For example, Table 4.2 contains repeated rows and repeated headings. Table 4.1 should be checked for consistency in units of measurement, years of measurement, and numerical presentation. Furthermore, the timeline structure between Table 4.1 and Table 4.2 (e.g., using the milestones 2010, 2015, 2020, 2023) is currently disjointed and lacks logical consistency, making it difficult for the reader to cross-reference the data accurately. The authors must indicate the sources of the GDP, HDI, PAPI, PCI, GII, DTI, and trust indicators, and when they were last updated.

 Additionally, the authors should avoid combining indicators with different scales or meanings without appropriate clarification for the reader. For example, PAPI scores, GII rankings, PCI rankings, and DTI scores are not directly comparable; therefore, the authors must define each variable, explain the rationale for its inclusion, and demonstrate how each variable supports the argument.

***The author acknowledges the limitation of the approach. However, for the purpose of providing evidence to the discussion, the authors have retained the tables, improved them and stated the prevailing limitations, indicating the respective literature gaps.***

***9. More precise articulation is needed regarding some of the claims***

 The authors use strong expressions (e.g., "soft resilience," "hard strength," "internal resource," and "regulatory system"), which are thought-provoking but require more precise definitions. The authors need to differentiate between metaphorical language, theoretical concepts, and empirically supported claims.

 To elaborate on the concept of grassroots democracy, the manuscript should demonstrate how principles such as "the people know, the people discuss, the people do, the people supervise, and the people benefit" support the authors' argument by providing concrete, identifiable, and verifiable examples. Currently, the manuscript introduces this principle somewhat abruptly without adequate introductory or transitional sentences. The authors should review the phrasing and context when presenting this principle to avoid disrupting the logical flow of the argument.

 The authors should avoid treating these concepts as mere statements of actual political and cultural change without providing real-world examples of their institutionalization, practice, and citizen affirmation.

***The authors have revised the respective sections as directed. Using words that can easily be understood by readers.***

**Other comments**

 The title and abstract are appropriate; the abstract will need to be updated following the resolution of methodological and data-related issues to reflect the research design and evidence accurately.

 Selected portions of the text can be condensed to reduce redundancy. Although the theoretical rationale is valuable, the text could be improved by a more structured organization.

 The writing is generally understandable; however, professional copyediting would help reduce academic jargon and clarify the meaning of long, overly undifferentiated sentences.

 All references must be thoroughly checked and verified for accuracy. Each in-text reference should correspond to a complete, matching reference. Any inconsistencies or errors in reference numbering should be resolved before publication.

 The authors should clarify whether this article represents primarily a conceptual work, a literature review, a policy analysis, or an empirical study using secondary indicators. At present, it contains all four types of writing; however, the article does not explicitly identify the genre of writing.

***Respective sections have been cross-checked again for consistency with the revision.***

**Suggestions for improving the quality of the manuscript**

***Methodological and structural revisions:***

 Resolve the methodological contradiction regarding Table 4.3: The authors must either provide comprehensive methodological and ethical details for the "Author's sociological survey, 2025," or remove it entirely. The Data Availability statement must be updated to reflect this decision.

 Refine causal claims and interpretations: The authors should explicitly frame indicators like GDP, HDI, and PAPI as indirect rather than definitive evidence of political culture change. Furthermore, claims regarding COVID-19 compliance and soft power should present political culture as one contributing factor among many, avoiding overly deterministic causal language.

 Extensive theoretical material on Marxism-Leninism and Ho Chi Minh Thought, currently located in the Results and Discussion section, should be moved to the Theoretical Framework. It will reduce redundancy and clarify the actual empirical findings.

 Correct data presentation in tables: Table 4.2 (which contains repeated rows and headings) and Table 4.1 (which requires consistency in measurement units and years) must be thoroughly revised. Clear sources, parameters, and explanations must be provided when combining composite indices like PAPI, PCI, GII, and DTI.

# Reviewer report: Reviewer response for version 1

**DOI:** 10.5256/f1000research.196055.r485086

**Published:** 2026-05-27

**Authors:** Vivi Indra Amelia Nasution

**Affiliations:** 1 Faculty of Law, Social, and Political Sciences, Universitas Terbuka, South Tangerang, Banten, Indonesia

Overall, this manuscript provides a well-structured and conceptually grounded documentary analysis, offering a valuable projection of Vietnam's political culture toward its 2045 national vision by tracking its evolution during the Doi Moi period across structural, behavioral, and institutional dimensions. The integration of secondary datasets offers a useful descriptive overview of the country’s socio-economic and digital transformation. However, several analytical issues require further attention.

 First, the connection between macro performance indicators and individual civic orientations warrants a more cautious interpretation. Since the study relies exclusively on qualitative content analysis and secondary data, the current argument risks drawing conclusions about individual civic behavior from aggregate institutional performance scores, creating a potential ecological fallacy. To maintain consistency with the political culture frameworks cited (Almond and Verba, Pye, Putnam), concepts such as “proactive citizenship” should be explicitly contextualized within the structural constraints of a highly centralized institutional setting.

 Second, the documentary analysis would benefit from a more balanced engagement with the evidence, as major governance transitions are rarely linear. Incorporating discussions of grassroots implementation frictions, institutional constraints, or real-world governance complexities—such as the bureaucratic anxieties accompanying large-scale anti-corruption campaigns—would provide a more nuanced and analytically grounded assessment.

 Third, the empirical narrative should be strengthened by complementing current performance-oriented metrics with datasets that track broader structural boundaries of civil society and civil liberties, such as those from the Varieties of Democracy (V-Dem) Institute or Freedom House. Using a broader range of metadata will prevent analytical conclusions from sounding overly optimistic based primarily on administrative or developmental successes.

 Finally, while the domestic focus provides a rich context for the case study, a limited comparative reflection would further enhance the manuscript’s broader scholarly contribution. The projected trends share observable structural patterns with governance adaptations across other regional single-party states. Acknowledging this comparative context and tempering absolute claims would strengthen the final section as a more analytically balanced analysis of state resilience in the 21st century.

Is the work clearly and accurately presented and does it cite the current literature?

Yes

If applicable, is the statistical analysis and its interpretation appropriate?

Not applicable

Are all the source data underlying the results available to ensure full reproducibility?

Partly

Is the study design appropriate and is the work technically sound?

Yes

Are the conclusions drawn adequately supported by the results?

Partly

Are sufficient details of methods and analysis provided to allow replication by others?

Partly

Reviewer Expertise:

International Relations, Comparative Politics, ASEAN Regional Politics, Public Policy, Comparative Governance, and Institutional Analysis

I confirm that I have read this submission and believe that I have an appropriate level of expertise to confirm that it is of an acceptable scientific standard, however I have significant reservations, as outlined above.

# Response

**Published:** 2026-06-27

**Authors:** QUOC KHANH LAI

**Affiliations:** Not Applicable, Vietnam

First, the connection between macro performance indicators and individual civic orientations warrants a more cautious interpretation. Since the study relies exclusively on qualitative content analysis and secondary data, the current argument risks drawing conclusions about individual civic behavior from aggregate institutional performance scores, creating a potential ecological fallacy. To maintain consistency with the political culture frameworks cited (Almond and Verba, Pye, Putnam), concepts such as “proactive citizenship” should be explicitly contextualized within the structural constraints of a highly centralized institutional setting.

***The respective sections have been revised, especially contextualizing the concepts of proactive citizenship to ensure caution.***

 Second, the documentary analysis would benefit from a more balanced engagement with the evidence, as major governance transitions are rarely linear. Incorporating discussions of grassroots implementation frictions, institutional constraints, or real-world governance complexities—such as the bureaucratic anxieties accompanying large-scale anti-corruption campaigns—would provide a more nuanced and analytically grounded assessment.

***The manuscript has been revised accordingly to factor in this comment.***

 Third, the empirical narrative should be strengthened by complementing current performance-oriented metrics with datasets that track broader structural boundaries of civil society and civil liberties, such as those from the Varieties of Democracy (V-Dem) Institute or Freedom House. Using a broader range of metadata will prevent analytical conclusions from sounding overly optimistic based primarily on administrative or developmental successes.

***The manuscript’s analysis section was revised to improve the analytical conclusions. Methodological limitations were stated since based on the subject of the study, getting coherent and consistent, and updated, comparable metadata for the analysis was a challenge.***

 Finally, while the domestic focus provides a rich context for the case study, a limited comparative reflection would further enhance the manuscript’s broader scholarly contribution. The projected trends share observable structural patterns with governance adaptations across other regional single-party states. Acknowledging this comparative context and tempering absolute claims would strengthen the final section as a more analytically balanced analysis of state resilience in the 21st century.

***The respective sections of the manuscript have been revised to strengthen the analytical vigor of the study.***

# Reviewer report: Reviewer response for version 1

**DOI:** 10.5256/f1000research.196055.r485094

**Published:** 2026-05-19

**Authors:** Nguyen Giang

**Affiliations:** 1 Ho Chi Minh University of Social Sciences and Humanities, Ho Chi Minh City, Vietnam

The author firmly asserts that the study is “entirely based on document analysis and secondary data sources,” “did not conduct any primary empirical research... such as surveys, interviews,” and therefore “is exempt from oversight by the institutional review board.” In section 4.3, the author unexpectedly includes Table 4.3 with a clearly cited source: “Author’s sociological survey, 2025” and analysis “Survey data (2025) shows...” This is a serious structural and logical inconsistency. If the paper used primary survey data conducted by the author in 2025, then the entire commitment and argument in Part III is completely empty and misleading.

 In Part II, the author constructed a “Theoretical Overview and Ideological Foundation,” analyzing Marxism-Leninism and Ho Chi Minh Thought. In Part IV (Results and Discussion), subsections 1 and 2 repeat the title and content: “The approach... of Marxist-Leninist and Ho Chi Minh thought” and “An integrated approach...”. Including purely theoretical discussion in the "Results" section is structurally incorrect. The Results section should present new findings from the data, not continue the interpretation of classical texts. This makes the article convoluted and repetitive in its conceptual structure.

 The article completely fails to demonstrate any specific mechanism of impact or causal/correlational relationship between changes in technical and economic indicators (DTI, GDP) and the cognitive nature of "political culture." The argumentative structure here is broken due to a lack of supporting evidence.

 The text explains that GDP per capita increased from $98 in 1990 to $402 in 2000. However, in Table 4.1, the figure for 2000 is recorded as "-402".

 Tables 4.1 and 4.2 are very disjointedly structured; the time milestones (2010, 2015, 2020, 2023) and figures (PAPI, HDI, GII, PCI) are scrambled and repeated illogically, making it impossible for the reader to accurately compare the data in the tables with the arguments presented by the author in the text.

 The article proposes a three-level analytical framework for political culture: (1) values ​​and ideologies, (2) norms and behaviors, and (3) institutions and governance culture. However, the definitions of levels 2 and 3 are unclear, indistinct, and lack persuasive structural system support.

 The author argues that high social compliance during the COVID-19 pandemic is evidence that political culture acts as a "social regulatory tool." This argument is flawed because it completely ignores other factors (such as fear of the disease, strict administrative sanctions, and legal pressure) and attributes the result solely to the power of "political culture."

 The author abruptly states: "From this perspective, this model shows that the principle often expressed as 'people know, people discuss, people know' is incorrect." However, the author fails to provide any preceding or following sentence to explain why this familiar principle of grassroots democracy is considered "incorrect," making the argument arbitrary and difficult to understand.

Is the work clearly and accurately presented and does it cite the current literature?

Partly

If applicable, is the statistical analysis and its interpretation appropriate?

Partly

Are all the source data underlying the results available to ensure full reproducibility?

Yes

Is the study design appropriate and is the work technically sound?

Partly

Are the conclusions drawn adequately supported by the results?

Yes

Are sufficient details of methods and analysis provided to allow replication by others?

No

Reviewer Expertise:

Asia Pacific political culture

I confirm that I have read this submission and believe that I have an appropriate level of expertise to confirm that it is of an acceptable scientific standard, however I have significant reservations, as outlined above.

# Response

**Published:** 2026-06-27

**Authors:** QUOC KHANH LAI

**Affiliations:** Not Applicable, Vietnam

The author firmly asserts that the study is “entirely based on document analysis and secondary data sources,” “did not conduct any primary empirical research... such as surveys, interviews,” and therefore “is exempt from oversight by the institutional review board.” In section 4.3, the author unexpectedly includes Table 4.3 with a clearly cited source: “Author’s sociological survey, 2025” and analysis “Survey data (2025) shows...” This is a serious structural and logical inconsistency. If the paper used primary survey data conducted by the author in 2025, then the entire commitment and argument in Part III is completely empty and misleading.

***This is revised as directed. Section 4.3 contains a table that has been extracted from the datasets from world bank, UNDP and Edelman Trust Barometer to show how macro indicators reflect the developmental context of Vietnamese political culture. These are secondary data sources.***

 In Part II, the author constructed a “Theoretical Overview and Ideological Foundation,” analyzing Marxism-Leninism and Ho Chi Minh Thought. In Part IV (Results and Discussion), subsections 1 and 2 repeat the title and content: “The approach... of Marxist-Leninist and Ho Chi Minh thought” and “An integrated approach...”. Including purely theoretical discussion in the "Results" section is structurally incorrect. The Results section should present new findings from the data, not continue the interpretation of classical texts. This makes the article convoluted and repetitive in its conceptual structure.

***This section has been revised accordingly, and the repetition has been removed. The results section presents new findings from literature and does not restate the theoretical framework.***

 The article completely fails to demonstrate any specific mechanism of impact or causal/correlational relationship between changes in technical and economic indicators (DTI, GDP) and the cognitive nature of "political culture." The argumentative structure here is broken due to a lack of supporting evidence.

***The section has been revised accordingly, providing supporting evidence, distinguishes between direct measures and indirect indicators, and acknowledges methodological limitations.***

 The text explains that GDP per capita increased from $98 in 1990 to $402 in 2000. However, in Table 4.1, the figure for 2000 is recorded as "-402".

***This section has been revised accordingly.***

 Tables 4.1 and 4.2 are very disjointedly structured; the time milestones (2010, 2015, 2020, 2023) and figures (PAPI, HDI, GII, PCI) are scrambled and repeated illogically, making it impossible for the reader to accurately compare the data in the tables with the arguments presented by the author in the text.

***The tables have been revised accordingly. However, since the table is informed by multiple sources or references, some limitations such as consistency in years are inevitable. Hence a slight variance in years between table 1 and table 2 (The tables have been renamed, from 4.1 to 1 and 4.2 to 2).***

 The article proposes a three-level analytical framework for political culture: (1) values ​​and ideologies, (2) norms and behaviors, and (3) institutions and governance culture. However, the definitions of levels 2 and 3 are unclear, indistinct, and lack persuasive structural system support.

***Based on the revisions earlier done for the paper, the author has improved on the analytical frameworks for levels 2 and 3.***

 The author argues that high social compliance during the COVID-19 pandemic is evidence that political culture acts as a "social regulatory tool." This argument is flawed because it completely ignores other factors (such as fear of the disease, strict administrative sanctions, and legal pressure) and attributes the result solely to the power of "political culture."

***The passage has been revised accordingly, acknowledged that political culture most likely acted as a social regulatory tool, bearing in mind that other factors may have also played a role.***

 The author abruptly states: "From this perspective, this model shows that the principle often expressed as 'people know, people discuss, people know' is incorrect." However, the author fails to provide any preceding or following sentence to explain why this familiar principle of grassroots democracy is considered "incorrect," making the argument arbitrary and difficult to understand.

***The section has been revised accordingly.***
